# VBayesMM: variational Bayesian neural network to prioritize important relationships of high-dimensional microbiome multiomics data

**DOI:** 10.1093/bib/bbaf300

**Published:** 2025-07-04

**Authors:** Tung Dang, Artem Lysenko, Keith A Boroevich, Tatsuhiko Tsunoda

**Affiliations:** Laboratory for Medical Science Mathematics, Department of Biological Sciences, School of Science, The University of Tokyo, 7-3-1 Hongo, Bunkyo-ku, Tokyo 113-0033, Japan; Laboratory for Medical Science Mathematics, Department of Biological Sciences, School of Science, The University of Tokyo, 7-3-1 Hongo, Bunkyo-ku, Tokyo 113-0033, Japan; Laboratory for Medical Science Mathematics, RIKEN Center for Integrative Medical Sciences, 1-7-22 Suehiro-cho, Tsurumi, Yokohama 230-0045, Japan; Laboratory for Medical Science Mathematics, RIKEN Center for Integrative Medical Sciences, 1-7-22 Suehiro-cho, Tsurumi, Yokohama 230-0045, Japan; Laboratory for Medical Science Mathematics, Department of Biological Sciences, School of Science, The University of Tokyo, 7-3-1 Hongo, Bunkyo-ku, Tokyo 113-0033, Japan; Laboratory for Medical Science Mathematics, RIKEN Center for Integrative Medical Sciences, 1-7-22 Suehiro-cho, Tsurumi, Yokohama 230-0045, Japan; Laboratory for Medical Science Mathematics, Department of Computational Biology and Medical Sciences, Graduate School of Frontier Sciences, The University of Tokyo, 7-3-1 Hongo, Bunkyo-ku, Tokyo 113-0033, Japan

**Keywords:** Bayesian neural network, variable selection, variational inference, integrative analysis, mouse and human microbiome multiomics data

## Abstract

The analysis of high-dimensional microbiome multiomics datasets is crucial for understanding the complex interactions between microbial communities and host physiological states across health and disease conditions. Despite their importance, current methods, such as the microbe–metabolite vectors approach, often face challenges in predicting metabolite abundances from microbial data and identifying keystone species. This arises from the vast dimensionality of metagenomics data, which complicates the inference of significant relationships, particularly the estimation of co-occurrence probabilities between microbes and metabolites. Here we propose the variational Bayesian microbiome multiomics (VBayesMM) approach, which aims to improve the prediction of metabolite abundances from microbial metagenomics data by incorporating a spike-and-slab prior within a Bayesian neural network. This allows VBayesMM to rapidly and precisely identify crucial microbial species, leading to more accurate estimations of co-occurrence probabilities between microbes and metabolites, while also robustly managing the uncertainty inherent in high-dimensional data. Moreover, we have implemented variational inference to address computational bottlenecks, enabling scalable analysis across extensive multiomics datasets. Our large-scale comparative evaluations demonstrate that VBayesMM not only outperforms existing methods in predicting metabolite abundances but also provides a scalable solution for analyzing massive datasets. VBayesMM enhances the interpretability of the Bayesian neural network by identifying a core set of influential microbial species, thus facilitating a deeper understanding of their probabilistic relationships with the host.

## Introduction

Recent studies have shown that shifts in the composition of the human gut microbiome significantly influence overall health [1, 2] and contribute to the development of various diseases such as inflammatory bowel disease [3] and cancer [4–7]. By leveraging advanced metagenomic sequencing techniques such as 16S rRNA sequencing and shotgun metagenomics, scientists can identify taxonomic units, often individual microbial species, linked to various diseases and investigate their functional roles within microbial communities [8–10]. The integration of diverse omics technologies—metagenomics, transcriptomics, and metabolomics—further enriches our understanding of the dynamic interactions between the host and its microbiome in human health and disease. Gut metabolites are primarily produced or influenced by microbial enzymes, which metabolize dietary elements and substances secreted by the host. Key microbial metabolites, such as short-chain fatty acids (SCFAs) and bile acids, serve as vital indicators of microbial fermentation and metabolic processes [11, 12]. For instance, the combination of low colonic SCFAs and high bile acids correlates with an increased risk of colon cancer among Americans consuming diets high in fats and proteins but low in complex carbohydrates [13]. Thus, the combined application of metagenomics and metabolomics presents a promising approach to decipher the complex biochemical interactions between the gut microbiota and the host [14, 15].

To explore the integration of disparate omics data, particularly the combination of microbial sequencing with mass spectrometry technologies for metabolites, researchers have developed several integrative analysis methods. However, current approaches encounter notable challenges when handling multiomics data. One primary issue arises from the differing measurement scales between sequencing and mass spectrometry, which complicates the analysis of relationships between microbiomes and metabolites due to the need for scale invariance [16, 17]. Traditional analytical methods, such as canonical correspondence analysis and sparse partial least squares (sPLS) regression [18, 19], struggle to maintain scale invariance when applied to interrelated datasets, primarily due to their underlying assumption of independent relationships. This limitation can lead to potential inaccuracies when combining microbiome and mass spectrometry data [16]. Furthermore, integrating these technologies produce high-dimensional and compositional datasets, making it challenging to discern biologically meaningful connections among the vast array of diverse biological variables. To tackle these challenges, neural network-based approaches have been developed to estimate co-occurrence probabilities between microbes and metabolites, offering a more robust solution for capturing complex, nonlinear relationships in multiomics data. The microbe–metabolite vectors (MMvec) approach, implemented in QIIME 2, is one of the most widely used methods for exploring microbiome–metabolite relationships [16, 20]. MMvec employs neural networks to calculate the conditional probabilities of detecting specific metabolites given the presence of particular microbes, thus addressing the limitations of previous methods that treated microbe–metabolite relationships as independent. This approach more effectively captures the complex dependencies in the data. Additionally, the neural network architecture of MMvec is specifically designed to handle the compositional nature of microbiome and mass spectrometry datasets. Traditional methods often struggle with inconsistencies between absolute and relative abundances across these datasets. By ensuring consistency between these two abundances, MMvec effectively reduces the false discovery rates [16]. The practical significance of the MMvec approach is well-documented, with multiple applications in human health and disease [21–23], environmental studies [24], and animal microbiome [25, 26], highlighting its utility in exploring the potential interrelations between microbiomes and metabolites across diverse fields.

However, the vast dimensionality of microbial metagenomics datasets poses significant challenges for the MMvec approach. Within this framework, all taxonomic units are treated with equal importance when computing co-occurrence probabilities between microbes and metabolites. This assumption, however, may not hold in practice, as a large number of taxonomic units might be irrelevant and thus may not contribute meaningfully to the identification or characterization of representative microbe–metabolite relationships. Emerging methods have proposed advanced architectures for enhancing the exploration of microbe–metabolite relationships. For instance, MiMeNet (Microbiome-Metabolome Network) [27] and mNODE (metabolomic profile predictor using neural ordinary differential equations) [28] employ multilayer perceptron (MLP) architectures with distinct computational strategies. While MiMeNet primarily uses standard MLP approaches, mNODE introduces a neural ordinary differential equation module placed between the hidden layers, enabling different mathematical operations. These methods, which utilize regularization techniques like $L_{2}$ regularization to address issues of sparsity [29], still face challenges in optimizing hyperparameters and in providing interpretable context for selected taxonomic units. Thus, there is still a definite need for an interpretable selection of key representative taxonomic units and probabilistically inferring which types of metabolites they are associated with. Furthermore, while the maximum a posteriori (MAP) probability estimates used in MMvec and standard approaches such as the sPLS and MiMeNet methods provide valuable point estimates for weight matrices and bias vectors, they do not sufficiently capture the uncertainty inherent in such predictive models, which can result in overconfident predictions that are ultimately inaccurate. These inaccuracies can result in valuable experimental resources being wasted on investigating ineffective or erroneous compounds [30–32]. Therefore, incorporating a reliable representation of uncertainty in predictive models is crucial to mitigate these risks. Recently, simulation approaches such as Markov chain Monte Carlo methods have been explored to improve uncertainty quantification in deep learning models, which could potentially enhance the reliability of their predictions [33, 34]. However, the direct applications of these approaches to microbiome multiomics studies present significant challenges due to the computational burden and the difficulty of achieving convergence in such complex high-dimensional datasets [35, 36].

In this study, we propose VBayesMM (variational Bayesian microbiome multiomics), a Bayesian neural network designed to address these limitations and enhance computational analysis scalability for large microbiome multiomics datasets. The main contributions of this study are three-fold. First, VBayesMM aims to improve the prediction of metabolite abundances from microbial metagenomics data by incorporating a spike-and-slab prior [37–39]. The trained model generates embeddings of microbial species and metabolites that are used to estimate co-occurrence probabilities between microbes and metabolites. VBayesMM can probabilistically identify a minimal core set of microbial species that significantly contribute to these predictions, enabling potential applications in the study of human microbiome-related diseases.

Second, the VBayesMM framework is structured as a variational Bayesian neural network [40, 41], which captures the uncertainties inherent in microbiome and metabolite datasets. This probabilistic framework allows us to calculate the standard deviation of the posterior probabilities of weight matrices and bias vectors, effectively quantifying predictive uncertainty.

Third, VBayesMM implements variational inference [42, 43] to overcome the computational inefficiencies traditionally associated with Bayesian methods when applied to high-dimensional input data. Variational inference has proven to be effective across a wide range of applications, including the analysis of large datasets related to metagenomics [44, 45], population genetics [46], and single-cell experiments [47–49].

Finally, to validate the performance of VBayesMM, we tested our approach on four public microbiome datasets paired with host metabolome data [16, 50–52]. These datasets cover diverse experimental conditions and profiling methods, including both 16S rRNA gene amplicon and whole-genome shotgun sequencing (WGS), paired with mass spectrometry-based metabolomics. The datasets vary substantially in size and complexity, ranging from hundreds to tens of thousands of taxonomic units, providing a comprehensive evaluation framework for our method. Further details about these datasets are included in the Data acquisition subsection of Materials and Methods.

## Materials and methods

### Overview of VBayesMM approach

**Figure 1:**
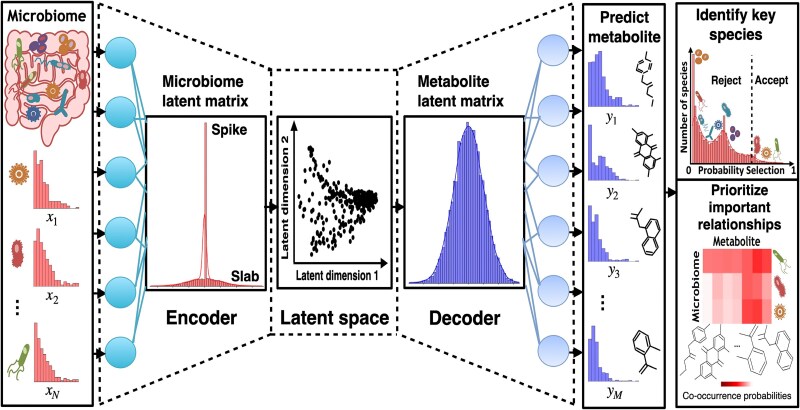
VBayesMM neural network architecture. VBayesMM uses paired microbiome–metabolite data, with microbial species as input variables and metabolite abundances as target variables. VBayesMM encodes input microbial sequence counts (x) (using neural network encoder) into a lower dimensional latent space that is utilized by the decoder to estimate conditional probabilities of observing specific metabolite abundances (y). VBayesMM implements a variational spike-and-slab distribution within the microbiome latent matrix to improve the probabilistic identification of microbial species, aiming to enhance predictive accuracy. A subset of microbial species, selected based on probabilistic thresholds, is then integrated with the metabolite latent matrix to explore microbiome–metabolite relationships. These relationships can be measured and ranked by the co-occurrence probabilities. Variational inference and stochastic optimization techniques are applied to effectively manage the computational challenges posed by the vast dimensionality of the input data.

The proposed VBayesMM approach (Fig. 1) builds on our previously introduced variational Bayesian method [42], which was highly effective for the tasks of microbiome clustering [44] and analysis of microbiome–metabolite relationships [45]. As shown in Fig. 1, the encoder component of the neural network models the latent space probability distribution conditioned on the observed microbiome input datasets, including sequence counts derived from either 16S rRNA gene amplicon or WGS. The 16S rRNA datasets are processed with the QIIME2 [20] to cluster sequences into operational taxonomic units (OTUs) or amplicon sequence variants. The WGS datasets are processed with the Kraken tool [53] that uses a k-mer-based approach to assign sequencing reads to known reference genomes. By learning a probabilistic mapping from these high-dimensional counts to a lower dimensional latent representation, the encoder can capture the underlying structure across multiple microbial taxa. To isolate a small subset of taxonomic units that enhance the accuracy and relevance of these predictions, we propose using a variational spike-and-slab distribution within the encoder neural network (applied to the weight matrix and bias vector). This model structure enables the differentiation of microbial taxa by employing a slab component to probabilistically highlight taxa with a potentially significant impact, while the spike component minimizes the influence of taxa with lesser or negligible impact on model performance (Fig. 1) [37–39].

In the second part of VBayesMM, the decoder segment generates conditional distributions for predicting metabolite abundances from the latent representation estimated during the encoding phase (Fig. 1). Like the MMvec approach, the metabolite abundances are modeled using a multinomial distribution. This architecture is specifically designed to systematically associate microbial taxa with metabolite abundances on a probabilistic basis, thereby enabling more accurate and interpretable predictions. Moreover, VBayesMM prioritizes small subsets of microbial species with the highest probability to be related to specific metabolite profiles (Fig. 1). These relationships are elucidated by the calculated co-occurrence probabilities.

### The architecture of the MMvec neural network

First, we provide a concise overview of the microbe–metabolite vectors (MMvec) approach, which estimates co-occurrence probabilities to elucidate the relationships between microbiomes and metabolites [16]. This method enables the determination of metabolite abundance data via conditional distributions based on microbiome abundances, utilizing the multinomial distribution. The fundamental concepts of the MMvec approach are as follows:

Given the paired microbiome-metabolome dataset $\mathbf{D} = \{ \mathbf{X}, \mathbf{Y} \}$, which includes K samples, N taxonomic units, and M metabolite abundances, we denote the observations for the $i$th taxonomic unit and the $j$th metabolite in the $k$th sample as $X_{ki}$ and $Y_{kj}$, respectively, where $k \in \{ 1, \ldots , K \}$, $i \in \{ 1, \ldots , N \}$ and $j \in \{1, \ldots , M \} $. The MMvec approach maps taxonomic units and metabolite abundances into a latent low-dimensional space of dimension L. Here, each $i$th taxonomic unit is represented by the vector $\mathbf{u}_{i} \in \mathbb{R}^{L}$, and each $j$th metabolite abundance by the vector $\mathbf{v}_{j} \in \mathbb{R}^{L}$. These latent vectors are drawn from a prior normal distribution with a mean of 0 and a diagonal covariance matrix $\text{I}$, where the variances for the taxonomic units and metabolites are $\sigma _{i}$ and $\sigma _{j}$, respectively:


\begin{align*} \mathbf{u}_{i} \sim \mathcal{N}(0,\sigma_{i}\text{I}) \\[1mm] \mathbf{v}_{j} \sim \mathcal{N}(0,\sigma_{j}\text{I}) \end{align*}

For a given microbial sample $\mathbf{X}_{k} = (X_{k1}, \ldots , X_{kN})$, each $i$th taxonomic unit is sampled from a categorical distribution, denoted as $i \sim \text{Categorical}(\mathbf{X}_{k})$. Given sample $k$, the MMvec models the metabolite abundances $\mathbf{Y}_{k} = (Y_{k1}, \ldots , Y_{kM})$ as being drawn from a multinomial distribution as follows:


(1) \begin{align*}& \mathbf{Y}_{k} \sim \text{Multinomial} \left( \mathit{n}, \overrightarrow{\text{p}_{i}}\right),\end{align*}

where $n$ is the total metabolite abundances across sample k and $\overrightarrow{\text{p}_{i}} = \left [ \text{p}_{1i},...,\text{p}_{ji},...,\text{p}_{Mi} \right ]$ is the conditional probability distribution of observing $j$th metabolite abundance given that $i$th taxonomic unit is observed. This is defined as follows:


(2) \begin{align*}& p_{ji} = \frac{ \text{exp} \left( \mathbf{v}_{j} \mathbf{u}_{i} + v_{j0} + u_{i0} \right)}{ \sum_{m} \text{exp} \left( \mathbf{v}_{m} \mathbf{u}_{i} + v_{m0} + u_{i0} \right)},\end{align*}

where $v_{j0}$ and $u_{i0}$ are biases used to accurately approximate the conditional distribution of microbiome–metabolite relationships. To refine the estimates, MAP estimation is used to optimize the objective function with respect to the matrices $\mathbf{U}$ and $\mathbf{V}$ [16]. Specifically, $\mathbf{U} = \left [0, \mathbf{u}_{0}, \mathbf{u}_{1},...,\mathbf{u}_{N}\right ]\in \mathbb{R}^{N \times L}$ denotes the embedding for N taxonomic units (assuming $\mathbf{u}_{0}, \mathbf{u}_{1},...,\mathbf{u}_{N}$ are independent.), and $\mathbf{V} = \left [\mathbf{v}_{0}, 0, \mathbf{v}_{1},..., \mathbf{v}_{M} \right ] \in \mathbb{R}^{M-1 \times L}$ denotes the embedding for M metabolite abundances. The zero elements in matrices $\mathbf{U}$ and $\mathbf{V}$ serve as reference points in the L-dimensional embedding space, constraining the parameter space and establishing a fixed coordinate system. For a given microbial sample $\mathbf{X}_{k}$, we compute the conditional probability of the metabolite abundances $\mathbf{Y}_{k}$ by first mapping $\mathbf{X}_{k}$ to the latent space via $\mathbf{U}$, and then using $\mathbf{V}$ to generate the corresponding metabolite predictions. This optimization is done using the Adam algorithm [54]:


\begin{align*}& \mathcal{L} = \sum_{k=1}^{K} \sum_{i=1}^{N} \sum_{j=1}^{M} \sum_{l=1}^{L} \left[ \begin{aligned} \mathcal{N} \left( U_{il} \vert 0, \sigma_{\mathbf{u}_{i}} \right) + \mathcal{N} \left( V_{jl} \vert 0, \sigma_{\mathbf{v}_{j}} \right) \\ + \text{Multinomial} \left( \mathbf{Y}_{k} \vert \overrightarrow{\text{p}_{i}}\right) \end{aligned} \right] \end{align*}

### Variational Bayesian neural network approach

In the MMvec approach, all microbial species are initially assumed to equally contribute to the prediction of the complete metabolite abundances profile. However, this assumption is often not appropriate in microbiome–metabolite research [55, 56], as a large number of microbial species—potentially amounting to tens of thousands of taxonomic units—may have negligible relevance. In reality, only a limited number of microbial species are involved in relationships with metabolites, and these relationships impact the estimations of the co-occurrence probabilities between microbes and metabolites. To address this issue, we propose incorporating a spike-and-slab prior, a method from Bayesian analysis [37–39, 57], into the latent microbiome matrix **U** of Bayesian neural network. This approach aims to prioritize the core set of microbial taxonomic units within the MMvec neural network. Consequently, the prior distribution of $i$th taxonomic unit $\mathbf{u}_{i}$ can be defined as follows [37–39]:


(3) \begin{align*}& \begin{aligned} &p \left( \mathbf{u}_{i} \vert \boldsymbol{\gamma}_{i} \right) \sim \boldsymbol{\gamma}_{i} \mathcal{N}(0,\beta_{0\mathbf{u}_{i}}^{2}) + \left( 1 - \boldsymbol{\gamma}_{i} \right) \delta_{0} \\ &p \left( \boldsymbol{\gamma}_{i} \right) \sim \text{Bernoulli} \left( \lambda \right), \end{aligned}\end{align*}

where $\boldsymbol{\gamma }_{i} \in \mathbb{R}^{L}$ is a binary vector, such as $\boldsymbol{\gamma }_{i} = 1$ indicates that the $i$th taxonomic unit is important for estimating the conditional distribution of $j$th metabolite abundance in equation 2 and $\boldsymbol{\gamma }_{i} = 0$ denotes that the $i$th taxonomic unit is unimportant for learning the co-occurrence probabilities. $\delta _{0}$ denotes the Dirac spike concentrated at zero and $\lambda $ is the hyperparameter of prior Bernoulli distribution. The prior distribution of $j$th metabolite abundance $\mathbf{v}_{j}$ follows normal distribution $\mathcal{N}(0,\beta _{0\mathbf{v}_{j}}^{2})$. In this spike-and-slab framework, $\beta _{0\mathbf{u}_{i}}^{2}$ and $\beta _{0\mathbf{v}_{j}}^{2}$ represent the variance parameters of the Gaussian priors for $\mathbf{u}_{i}$ and $\mathbf{v}_{j}$, respectively. Although these parameters are functionally similar to the $\boldsymbol{\sigma }$ in the MMvec approach, we adopt the $\boldsymbol{\beta }$ notation to maintain consistency with the spike-and-slab prior convention.

We propose the Variational Bayesian (VB) method [37–39, 58] to perform efficient approximate posterior inference, which is necessary for learning the co-occurrence probabilities between the microbiome and metabolome. Our framework uses several key variables: $\boldsymbol{\Xi }$, which includes the embedding matrix for microbial species $\mathbf{U}$, a binary matrix for taxonomic unit selection $\boldsymbol{\gamma }$, and the embedding matrix for metabolite abundances $\mathbf{V}$. To manage the computational complexity and intractability of the true posterior distribution $p(\boldsymbol{\Xi } \vert \mathbf{D})$, we introduce a variational distribution $q(\boldsymbol{\Xi } \vert \Theta )$, characterized by the hyperparameter $\Theta $. This approach allows for a tractable and effective approximation of the posterior distribution. In VBayesMM, these variational distributions are defined as follows:


(4) \begin{align*}& \begin{aligned} &q \left(\mathbf{U}, \boldsymbol{\gamma}, \mathbf{V} \vert \Theta \right) = \prod_{i=1}^{N} \prod_{l=1}^{L} q \left( U_{il} \right) \times \prod_{i=1}^{N} \prod_{l=1}^{L} q \left( \gamma_{il} \right) \times \prod_{j=1}^{M} \prod_{l=1}^{L} q \left( V_{jl} \right), \end{aligned}\end{align*}

where


(5) \begin{align*}& \begin{aligned} &q \left( \mathbf{U} \right) \sim \boldsymbol{\gamma} \mathcal{N} \left(\boldsymbol{\alpha}_{\mathbf{U}},\boldsymbol{\beta}^{2}_{\mathbf{U}}\right) + \left( 1 - \boldsymbol{\gamma} \right) \delta_{0}\\ &q \left(\boldsymbol{\gamma} \right) \sim \text{Bernoulli} \left( \boldsymbol{\xi} \right)\\ &q \left( \mathbf{V} \right) \sim \mathcal{N} \left(\boldsymbol{\alpha}_{\mathbf{V}},\boldsymbol{\beta}^{2}_{\mathbf{V}}\right). \end{aligned}\end{align*}

In Equation 5, the variational parameter $\Theta $ comprises $\{ \boldsymbol{\alpha }_{\mathbf{U}},\boldsymbol{\beta }_{\mathbf{U}}, \boldsymbol{\xi }, \boldsymbol{\alpha }_{\mathbf{V}},\boldsymbol{\beta }_{\mathbf{V}} \}$. The distributions of the exponential families were selected for the variational distributions to guarantee a feasible computation of the expectations. The log marginal probability $\text{log} \left ( p \left ( \mathbf{D} \right ) \right )$, which is known as the evidence of **D**, is defined as follows:


(6) \begin{align*}& \begin{aligned} \text{log} \left( p \left( \mathbf{D} \right) \right) = \text{KL} \left[ q \left( \boldsymbol{\Xi} \vert \Theta \right) \| p \left( \boldsymbol{\Xi} \vert \mathbf{D} \right) \right] + \mathcal{L} \left[ q \left( \boldsymbol{\Xi} \vert \Theta \right) \right], \end{aligned}\end{align*}

where the first term of equation 6 is the KL divergence to measure the similarity between $q \left ( \boldsymbol{\Xi } \vert \Theta \right )$ and $p \left ( \boldsymbol{\Xi } \vert \mathbf{D} \right )$. Since $\text{KL} \left [ q \left ( \boldsymbol{\Xi } \vert \Theta \right ) \| p \left ( \boldsymbol{\Xi } \vert \mathbf{D} \right ) \right ] \geq 0$, the VB approach optimizes the second term of Equation 6, which is called evidence lower bound (ELBO), defined as follows:


(7) \begin{align*}& \begin{aligned} \mathcal{L} \left[ q \left( \boldsymbol{\Xi} \vert \Theta \right) \right] &= \text{E}_{q} \left[ \text{log} \left( p \left( \boldsymbol{\Xi}, \mathbf{D} \right) \right) \right] - \text{E}_{q} \left[ \text{log} \left( q \left( \boldsymbol{\Xi} \vert \Theta \right) \right) \right]\\ &= \text{E}_{q} \left[ \text{log} \left( p \left( \mathbf{D} \right) \right) \right] \\ & \quad - \text{KL} \left[ q \left(\boldsymbol{V} \right) \| p \left(\boldsymbol{V} \right) \right] - \text{KL} \left[ q \left(\boldsymbol{\gamma} \right) \| p \left(\boldsymbol{\gamma} \right) \right] \\ & \quad - q \left( \boldsymbol{\gamma} = 1 \right) \text{KL} \left[ \mathcal{N} \left(\boldsymbol{\alpha}_{\mathbf{U}},\boldsymbol{\beta}^{2}_{\mathbf{U}}\right) \| \mathcal{N} \left(0,\boldsymbol{\beta}^{2}_{0\mathbf{U}}\right) \right]. \end{aligned}\end{align*}

In equation 7, the variational parameters for the normal variables **U** and **V** are reparameterized using the expression $\boldsymbol{\alpha } + \boldsymbol{\beta } \boldsymbol{\epsilon }$, where $\boldsymbol{\epsilon }$ follows a standard normal distribution, $\mathcal{N}(0,1)$ [58]. Since the variable $\boldsymbol{\gamma }$, which represents microbial selection, is discrete, the continuous reparameterization trick is not applicable. To address this, we adopt the Gumbel-softmax approximation [37–39], which facilitates the reparameterization of categorical variables as follows:


\begin{align*}& \begin{aligned} &\overline{\boldsymbol{\gamma}} = \left( 1 + \text{exp} \left( - \frac{\boldsymbol{\zeta}}{\iota} \right) \right)^{-1}\\ &\boldsymbol{\zeta} = \text{log} \frac{\boldsymbol{\xi}}{ 1 - \boldsymbol{\xi}} + \text{log} \frac{\boldsymbol{\kappa}}{ 1 - \boldsymbol{\kappa}}, \end{aligned} \end{align*}

where $\boldsymbol{\kappa } \sim U \left ( 0, 1\right )$ and $\iota $, which represents the temperature parameter, is set to a minimum of 0.5 to ensure numerical stability [37–39]. Since the variational distribution $q(\boldsymbol{\Xi } \vert \Theta )$ is reparameterized into differentiable forms, we can utilize the stochastic gradient approach [54] to optimize the ELBO $\mathcal{L} \left [ q \left ( \boldsymbol{\Xi } \vert \Theta \right ) \right ]$. The mathematical details of the gradient of ELBO $\nabla _{\left ( \Theta \right )} \mathcal{L} \left [ q \left ( \boldsymbol{\Xi } \vert \Theta \right ) \right ]$ with respect to variational parameters are provided in the Supplementary Material. The complete procedure of VBayesMM is defined as follows:




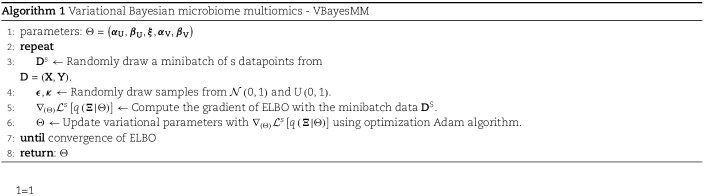



### Criteria to evaluate the performance of the approaches

In both the MMvec and VBayesMM approaches, the data are actual counts of metabolite abundances, so the predictive performance on test samples can be determined using mean absolute error (MAE). The MAE is calculated as follows [16]:


\begin{align*}& \begin{aligned} \text{MAE} = \frac{\sum_{k^{\prime}=1}^{K^{\prime}} \vert \mathbf{Y}_{k^{\prime}} - w_{k^{\prime}} \text{softmax} \left( \mathbf{V} \mathbf{U} \right) \vert}{K^{^{\prime}}}, \end{aligned} \end{align*}

where $w_{k^{\prime}}$ denotes the total metabolite abundances in test sample $k^{\prime} \in \{ 1,..., K^{^{\prime}}\}$ and |.| is the absolute value. For each microbial species, data are resampled from a categorical distribution based on the microbial composition of the testing sample $\mathbf{X}_{k^{\prime}}$. To validate performance, 20% of the total samples across all four datasets were randomly set aside as test samples, while the remaining 80% were used for training.

Then, in order to assess the performance of these approaches across datasets, which often exhibit significant variations in the scale of observations, we use the symmetric mean absolute percentage error (SMAPE) as a robust measure of accuracy [59]. This metric provides a reliable comparison regardless of the dataset size or scale and is computed as follows:


\begin{align*}& \begin{aligned} \text{SMAPE} = \frac{100}{K^{\prime}} \sum_{k^{\prime}=1}^{K^{\prime}} \frac{ \vert \mathbf{Y}_{k^{\prime}} - \hat{\mathbf{Y}}_{k^{\prime}} \vert}{\vert \mathbf{Y}_{k^{\prime}} \vert + \vert \hat{\mathbf{Y}}_{k^{\prime}} \vert}, \end{aligned} \end{align*}

where |.| is the absolute value, $\hat{\mathbf{Y}}_{k^{\prime}}$ is the predicted value, and $ 0\% \leqslant \text{SMAPE} \leqslant 100\% $.

### Data acquisition

We used microbiome–metabolite data from previously published datasets composed of both mouse and human samples. Dataset A is from a study on obstructive sleep apnea (OSA) in mice, including 92 samples for intermittent hypoxia and hypercapnia (IHH) and 90 samples for control conditions, along with 4702 taxonomic units and 362 metabolite abundances [50]. Metabolomics detection and annotation were performed using the Global Natural Product Social Molecular Networking platform (GNPS) [60]. This analysis identified a number microbe-dependent metabolites, such as bile acids (chenodeoxycholic acid, cholic acid), phytoestrogens (enterodiol, enterolactone), and fatty acids (elaidic acid, phytomonic acid), with differential abundances between the IHH and control groups [50]. Dataset B, from a study on effects of high-fat diet (HFD) in a murine model, has 434 samples, 913 taxonomic units, and 2257 metabolite abundances [16]. GNPS facilitated the determination of metabolites such as secondary bile acids, primary bile acids, soyasaponins, and peptides, which are microbially produced. Datasets A and B were analyzed using 16S rRNA gene sequencing-based microbiome and liquid chromatography-tandem mass spectrometry-based metabolome datasets.

Dataset C was generated from the fecal microbiome and metabolites profiles in gastric cancer (GC) patients, comprising of 42 samples of gastrectomy cases and 54 samples of healthy controls, with 48 243 taxonomic units and 183 metabolite abundances [52, 61]. The metabolites were summarized and annotated using the Kyoto Encyclopedia of Genes and Genomes (KEGG) [62]. This annotation process helped in the identification of key microbe-dependent metabolites such as glycocholate, taurine, and cholate, which were found to be significantly enriched in the gastrectomy group compared with controls [52]. Dataset D focuses on the fecal microbiome and metabolites in colorectal cancer (CRC) patients from stage 0 to stage 4, with 150 samples containing 57 702 taxonomic units and 169 metabolite abundances [51, 61]. KEGG annotation identified bacterial metabolites such as bile acids and SCFAs, which have established associations with the CRC stages. Datasets C and D have WGS microbiome profiling and capillary electrophoresis time-of-flight mass spectrometry for metabolomics.

### Open-source software

The VBayesMM package has been implemented using Tensorflow, the same framework used to develop MMvec, with an additional version available in PyTorch. This dual-framework compatibility allows users to select the most suitable version for their own analysis pipeline. The software accepts microbiome and metabolite count data in CSV, TSV, or BIOM formats. The primary outputs of both MMvec and VBayesMM include the microbiome matrix **U**, the metabolite matrix **V**, and a matrix of log conditional probabilities for the relationships between microbial species and metabolites. A unique feature of VBayesMM is the inclusion of a selection probabilities matrix for microbial taxonomic units, which is particularly valuable when dealing with large datasets. This matrix enables users to identify and focus on the most important taxonomic units that show significant relationships with metabolites, facilitating the construction of informative heatmaps. Additionally, the software provides the ELBO and MAE outputs to evaluate model performance.

Users can set the number of gradient descent iterations and batch size, which traditionally impact computational times. Our computations were conducted on an Intel$\circledR $ Xeon$\circledR $ Gold 6230 Processor, featuring 2.10 GHz $\times $ 2, with 40 cores and 2 threads per core, running Ubuntu 22.04.4 LTS. In scenarios involving dataset A, the runtime for VBayesMM, utilizing all 40 cores (equivalent to 80 logical processors), is typically around 1.5 h to achieve model convergence. Dataset B, which contains 2257 metabolite abundances, reached a convergence after running for about 8.5 h. For datasets with a large number of microbial taxonomic units, such as dataset C, which includes 48 243 microbial taxa and 183 metabolite abundances, convergence was reached within $\sim $2 days. Similarly, dataset D, which contains 57 702 microbial taxa and 169 metabolite abundances, requires about 5 days to converge. These timelines reflect the processing capacity and efficiency of our computational setup, allowing timely analysis despite the scale of the data. The details of computational time are provided in Supplementary Table S2.

In all our experiments, we set the number of latent dimensions to 3, the prior distributions of mean and standard deviation for the weight matrix and bias vector of microbial species **U** and metabolite abundances **V** that follow the standard normal distributions, $\mathcal{N} \left (0,1 \right )$. For the Adam algorithm, the learning rate is 0.1, the exponential decay rate for the first-moment estimates is 0.8, and the exponential decay rate for the second-moment estimates is 0.9 [16]. To address the selection of microbial species, we set the value of the temperature parameter $\iota $ to 0.5, the initial value of the hyperparameter $\xi $ of Bernoulli prior distribution to $U \left ( 0, 1\right )$, and the value of the hyperparameter $\lambda $ to $\text{log} \left ( \lambda ^{-1}\right ) = \text{log} \left ( (N+1) L \right ) + 0.1 \left ( 2 \text{log} (N) + \text{log}(\sqrt{K}N)\right ) $ [37]. Due to significant differences in the number of microbial features (N) and samples (K) across our datasets, the actual values of $\lambda $ differ for each dataset. This theoretically justified choice provides dataset-specific regularization that incorporates knowledge of the input dimension, network structure, and sample size, in contrast to the heuristic choices used in other methods. This adaptive approach ensures appropriate sparsity levels for each dataset, preventing overfitting in datasets with numerous microbial features.

## Results

### VBayesMM achieves accurate performance in large datasets

To evaluate the performance of VBayesMM, we applied it to four multiomics microbiome datasets derived from human and mouse samples (see Methods), which included thousands to tens of thousands of features such as microbiome species and metabolite abundances. We conducted a direct comparison using the default settings of the MMvec package and MiMeNet package in Python, and additionally employed the sPLS method from the mixOmics package in R, a tool designed for integrating multiple data types [18, 19]. The sPLS method was used with the default parameters of the *tune.spls* function, allowing us to compare the performance of VBayesMM, MiMeNet, MMvec, and sPLS across diverse datasets. The performance of each method was evaluated using the SMAPE, a reliable metric for assessing their effectiveness across datasets of varying scales.

**Figure 2:**
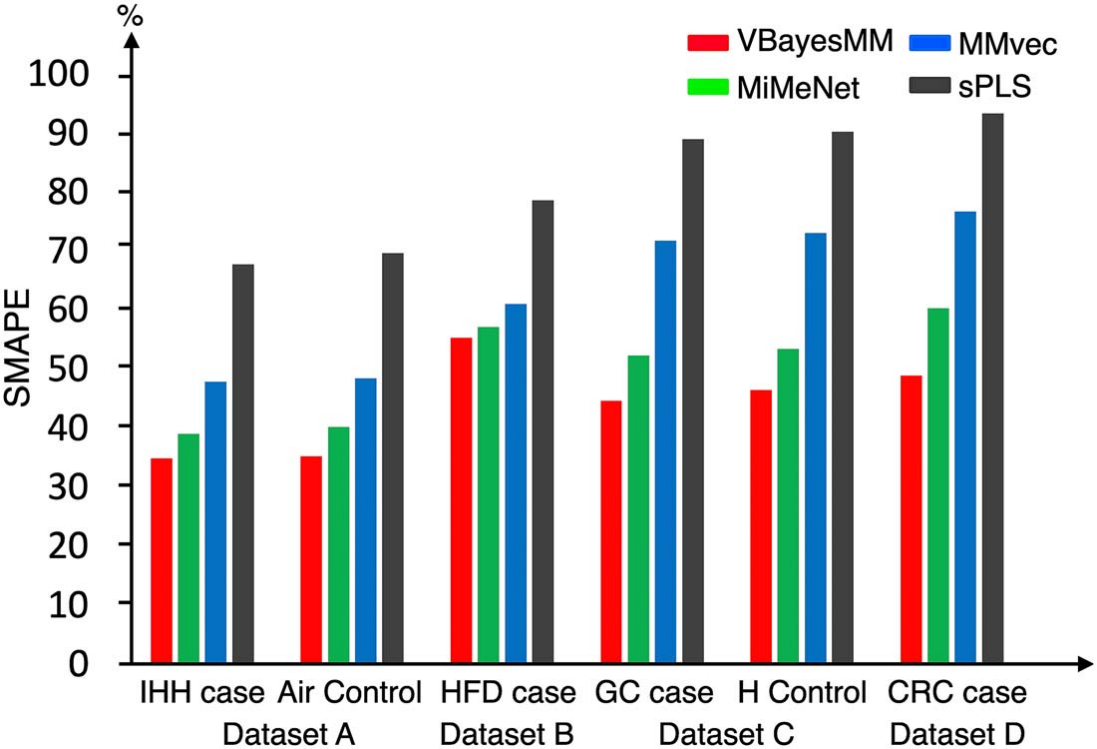
SMAPE values are computed for four approaches on real datasets. Dataset A includes cases of IHH and control. Dataset B includes an HFD case. Dataset C comprises a GC case and a healthy (H) control. Dataset D contains a CRC case. Note: All algorithms were run on a personal computer (Intel$\circledR $ Xeon$\circledR $ Gold 6230 Processor 2.10 GHz $\times $ 2, 40 cores, 2 threads per core) under Ubuntu 22.04.4 LTS.

The SMAPE values were used to assess the effectiveness of the VBayesMM method in comparison with MiMeNet, MMvec, and sPLS, as detailed in Fig. 2 and Supplementary Table S1. VBayesMM consistently achieved lower SMAPE values compared with MiMeNet, MMvec, and sPLS in all tested datasets. Specifically, in dataset A, VBayesMM achieved the lowest SMAPE values (34.74% for the case group and 35.05% for the control group). MiMeNet followed it with 38.82% and 40.11%, respectively, whereas MMvec yielded 47.59% and 48.17%. The three neural network-based methods performed better than the traditional sPLS approach, with SMAPE scores of 67.52% for the IHH cases and 69.37% for controls. As shown in Supplementary Table S2, MiMeNet ran to completion in just over 1.2 h, while VBayesMM and MMvec required $\sim $1.5 h, and sPLS taking the longest. Moreover, Supplementary Figure S1A supports these findings by illustrating the ELBO values for VBayesMM’s training and comparing the MAE values with MMvec during the validation phase. The graphical representations in Supplementary Figures S1Ac and S1Ad emphasize VBayesMM’s stability and lower MAE values *versus* the variable results of MMvec. In dataset B, where the number of metabolite abundances was notably higher at 2257 compared with 362 in dataset A, and the number of taxonomic units was substantially lower at 913 compared with 4702 in dataset A, the performance metrics indicated an increase in SMAPE values. Specifically, VBayesMM achieved the lowest SMAPE (55.07%), narrowly surpassing MiMeNet (56.88%) and outperforming MMvec (60.77%) and sPLS (78.32%). Nonetheless, MiMeNet finished faster (6.92 h) than both MMvec (8.28 h) and VBayesMM (8.54 h). Although this indicates that VBayesMM’s advantage in accuracy may come with a higher computational cost, particularly when metabolite abundances increase disproportionately to taxonomic units, it nevertheless outperforms the other methods.

To evaluate the efficacy of VBayesMM across different metagenomic sequencing methods, we used integrated shotgun metagenomic sequencing and mass spectrometry datasets. These datasets have higher complexity and dimensionality compared with 16S datasets [63, 64]. Specifically, dataset C is comprised of 48 243 taxonomic units, far exceeding those in datasets A and B. However, the number of metabolite abundances (183) is smaller than in datasets A (362) and B (2257). Figure 2 and Supplementary Table S1 show higher SMAPE values in dataset C compared with datasets A and B. VBayesMM achieved SMAPE values of 44.42% for the GC case and 46.31% for the healthy control, surpassing MiMeNet’s respective values of 52.03% and 53.81%. MMvec and sPLS showed higher SMAPE values, ranging from 71.58% to 90.03%. According to Supplementary Table S2, MiMeNet took around 39 h, whereas MMvec and VBayesMM required roughly 48 h. Additionally, Supplementary Figures S1Cc and S1Cd illustrate VBayesMM’s superior performance in both GC cases and health controls, displaying consistently lower and more stable MAE values, in contrast to the fluctuation observed with the MMvec approach.

Despite dataset D featuring the largest number of taxonomic units among the evaluated datasets, totaling 57 702, it had a relatively small number of metabolite abundances at just 169. The VBayesMM approach demonstrated improved performance for this dataset, achieving the lowest SMAPE (48.64%), while MiMeNet’s value was higher at 60.06%. MMvec and sPLS exhibited considerably larger SMAPE values, at 76.45% and 93.12%, respectively. MiMeNet continued to have the shortest runtime at around 98.51 h, whereas MMvec and VBayesMM exceeded 120 h. Moreover, Supplementary Figure S1Db illustrates that the MAE value for the VBayesMM was not only significantly lower but also more stable than the MAE value for the MMvec. Figure 2 shows that the neural network methods again performed better than the traditional method sPLS, particularly in the integrative analysis of shotgun metagenomics and multi-omics datasets. While VBayesMM required more computational time, these results show that it still offered relatively higher accuracy and stability when applied to ultra-high dimensional datasets than other tested approaches.

### VBayesMM identifies a core set of features for microbial species

**Figure 3:**
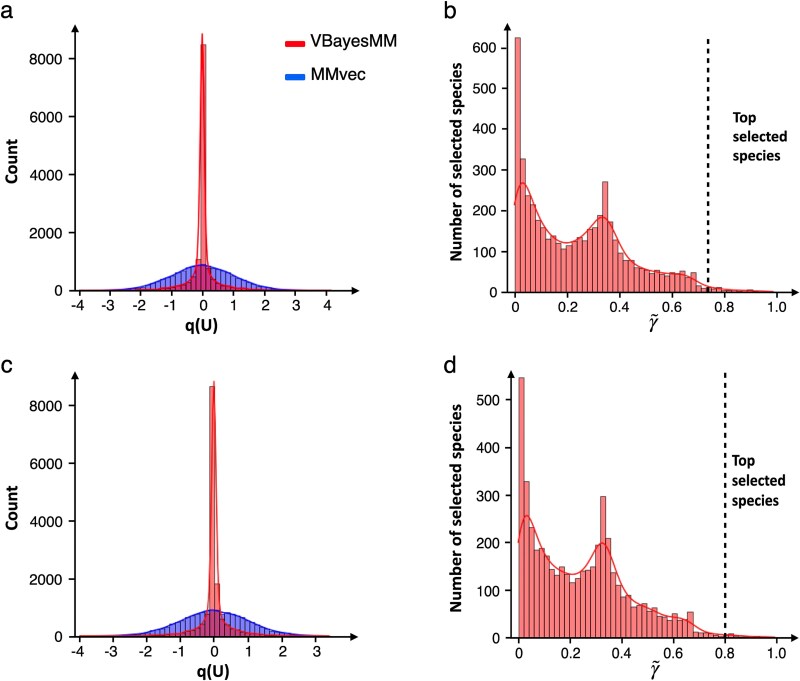
Histogram of the variational distribution $q \left (\mathbf{U}\right )$ and the average of $\boldsymbol{ \tilde \gamma } = \frac{\sum _{l=1}^{L} \gamma _{il}}{L}$ in dataset A. The dashed lines are bound to select microbiome species. (a-b) IHH cases group. (c-d) controls group.

To evaluate the effectiveness of VBayesMM in identifying keystone microbial species that improve the accuracy of co-occurrence probability estimates between microbes and metabolites, we used the variational distributions of the latent microbiome matrix **U** and microbial species selection (denoted as $\boldsymbol{\gamma }$). These distributions are shown in Figs 3a and 3c for dataset A, with Supplementary Figure S5a providing additional insights for dataset B.

Specifically, VBayesMM is better at removing the non-informative microbial species because it takes advantage of the spike-and-slab distribution. Unlike the normal distribution used in MMvec, it is characterized by a sharp peak at origin, which leads to most of the variable weights to be set to zero. The spike component of the distribution aggressively enforces sparsity, which is highly beneficial in this case as it aligns with the baseline assumption that most microbial species will not significantly contribute to the model. Conversely, the slab component of the distribution, represented by the distribution’s tails, retains a subset of microbial species deemed potentially important, allowing their contributions to be accurately estimated without being affected by substantial shrinkage.

In contrast, the normal distribution used in the MMvec approach, with its broader spread, implies a more uniform assignment of variable weights, making nonzero weights more likely. This can lead to noise and potentially irrelevant microbial species being included in the model. This advantage of the spike-and-slab approach is demonstrated in comparisons that were done for these two strategies, where we have shown improvements in distinguishing influential from non-influential species, and, consequently, relevant co-occurrence probability estimates.

To illustrate how VBayesMM identifies a minimal core set of microbial species, we used the histograms showing the average variational probability $\gamma _{il}$ across the latent dimension $l$th within dataset A, expressed as $\left ( \boldsymbol{\tilde \gamma } = \frac{\sum _{l=1}^{L} \gamma _{il}}{L} \right )$, as shown in Figs 3b and 3d. Notably, the probability of $\boldsymbol{ \tilde \gamma }$ peaks sharply near 0, indicating that a substantial number of species have a very low probability of being influential, and therefore are not selected. This strict enforcement of sparsity by VBayesMM systematically excludes species with small likelihood contributions, simplifying the model and facilitating identification of species with more significant impacts.

Another secondary peak at 0.35 suggests a threshold for potentially influential species, with probability decreasing markedly after this point, indicating fewer species passing the threshold beyond this level. The selection threshold is indicated by a dotted line, and any species above it are considered highly influential. For example, we identified the top 50 microbial species with $\boldsymbol{\tilde \gamma }$ values over 0.75 for IHH cases and over 0.8 for controls. Supplementary Figure S2 shows a scatter plot of the variational probability $\boldsymbol{ \tilde \gamma }$ for all microbial species in the IHH case and control groups. This visualization offers an overview of how taxonomic units differ between these two conditions. In addition, Supplementary Figures S5a and b show the variational distributions of **U** and the probability of $\boldsymbol{\tilde \gamma }$ in dataset B. Here, we selected the top 50 microbial species with $\boldsymbol{\tilde \gamma }$ values exceeding 0.7 in the HFD samples. The selection is notably nonuniform, prioritizing species with higher $\boldsymbol{\tilde \gamma }$ values. These figures illustrate the ways in which the proposed probability-based selection process can facilitate better understanding of how key microbial species contribute to the metabolite composition of the samples.

**Figure 4:**
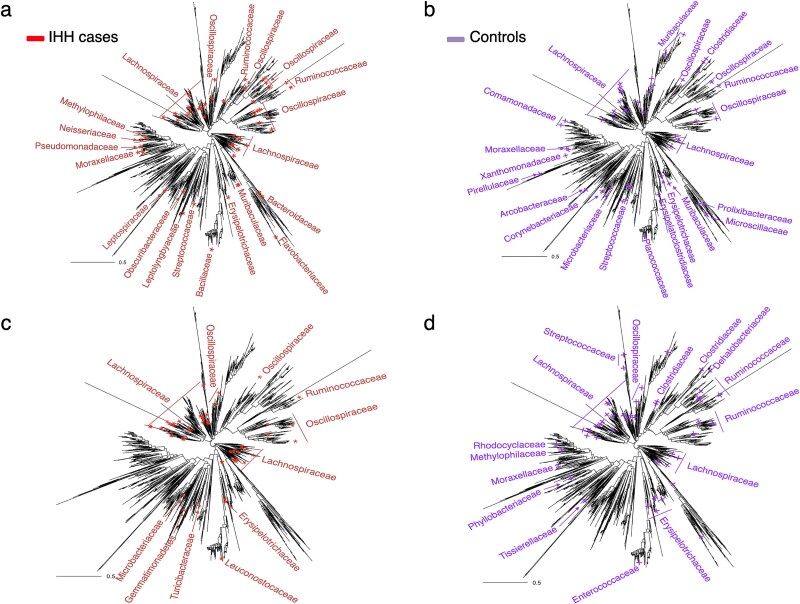
Microbial species selected using the VBayesMM and MMvec approaches and mapped on the phylogenetic tree based on 16S rRNA gene sequences for dataset A. (a-b) VBayesMM. (c-d) MMvec.

To confirm the biological significance of microbial species identified by VBayesMM in OSA study, we mapped the top 50 species associated with IHH cases and controls in dataset A onto the 16S phylogenetic tree, as shown in Figs 4a and 4b. Figures 4c and 4d provide comparable trees of the top 50 microbial species associated with IHH cases and controls, selected by MMvec via ranking the estimated microbe–metabolite co-occurrence probabilities. VBayesMM and MMvec identified species from the *Lachnospiraceae*, *Oscillospiraceae,* and *Ruminococcaceae* families within the *Bacillota* phylum as particularly relevant for the IHH condition (Fig. 4a and 4c). According to Supplementary Figure S2, these families also show high $\boldsymbol{\tilde \gamma }$ values (exceeding 0.85), due to their consistent presence across IHH samples. Similar observations were reported in previous works [50]. Several studies on OSA have reported an increased prevalence of *Lachnospiraceae* and *Ruminococcaceae*, which are recognized for their fermentative abilities and associations with systemic inflammation, adipose tissue changes, and shifts in insulin sensitivity. These changes in gut microbiota may affect metabolic health through mechanisms such as disruption of the colonic epithelial barrier [65, 66].

Additionally, families from the *Pseudomonadota* phylum, including *Methylophilaceae*, *Neisseriaceae*, *Pseudomonadaceae*, and *Moraxellaceae* appeared predominantly in IHH samples and exhibited $\boldsymbol{\tilde \gamma }$ values ranging from 0.78 to 0.84 (Fig. 4a and Supplementary Figure S2). By contrast, these families were less evident in the MMvec results (Fig. 4c). In the control group, MMvec distinctly detected species from the *Rhodocyclaceae*, *Phyllobacteriaceae*, and *Tissierellaceae* families (Fig. 4d), whereas VBayesMM identified families within the *Pseudomonadota* phylum, such as *Comamonadaceae* and *Xanthomonadaceae* families (Fig. 4b), which appeared at higher levels with $\boldsymbol{\tilde \gamma }$ values of 0.90 (Supplementary Figure S2). These observations by using VBayesMM align with prior studies [67–70]. For example, members of *Neisseriaceae* family are known for their ability to reduce nitrate to nitrite in the oral cavity, which can be absorbed and converted into nitric oxide, a blood pressure regulator. Previous studies have shown significant enrichment of these bacteria in the hypertensive group [67, 68], suggesting a link to inflammation and certain cardiovascular conditions, and highlighting their potential impact on systemic health through their metabolic functions.

Previous research has consistently emphasized the substantial impact of the *Gammaproteobacteria* class, including families such as *Pseudomonadaceae* and *Moraxellaceae*, on OSA [69, 70]. This bacterial class is closely associated with inflammatory processes within the human body, comprising multiple species directly involved in various pathological conditions. Notably, an increase in the *Gammaproteobacteria* class within the microbiome strongly correlates with elevated levels of pro-inflammatory cytokines, such as interleukin-6 [69]. This relationship highlights the critical role these bacteria play in exacerbating the inflammatory conditions observed in OSA, suggesting that their presence may significantly influence the severity and progression of the disease.

In the next application case, we applied the VBayesMM approach to determine the association of various microbial compositions with an HFD in dataset B. This top 50 critical microbial species identified are displayed on the 16S phylogenetic tree in Supplementary Figure S6. Supplementary Figure S7 provides comparable trees of the top 50 microbial species, selected by MMvec. The analysis highlighted keystone species from the *Lachnospiraceae*, *Oscillospiraceae*, and *Ruminococcaceae* families within the *Bacillota* phylum, as well as *Muribaculaceae*, *Rikenellaceae*, and *Marinifilaceae* families from the *Bacteroidota* phylum (Supplementary Figure S6). Recent studies indicate that HFDs induce a significant shift in gut microbiota, notably marked by an increased presence of *Lachnospiraceae*. This shift underscores their pivotal role in energy metabolism, which may be driving the increased adiposity and obesity observed in experimental models, while also emphasizing their substantial impact on metabolic health [71–73]. The pronounced abundance of *Lachnospiraceae* in response to high-fat dietary patterns strongly correlates with greater risks of metabolic disorders and diabetes, suggesting that these bacteria may play a crucial role in exacerbating the metabolic patterns associated with obesity [71]. Emerging evidence highlights the substantial role of dietary fats in modulating gut microbiota including its downstream effects on overall metabolic health and disease progression.

### VBayesMM prioritizes the co-occurrence probabilities between the most informative microbial species and metabolite abundances

**Figure 5:**
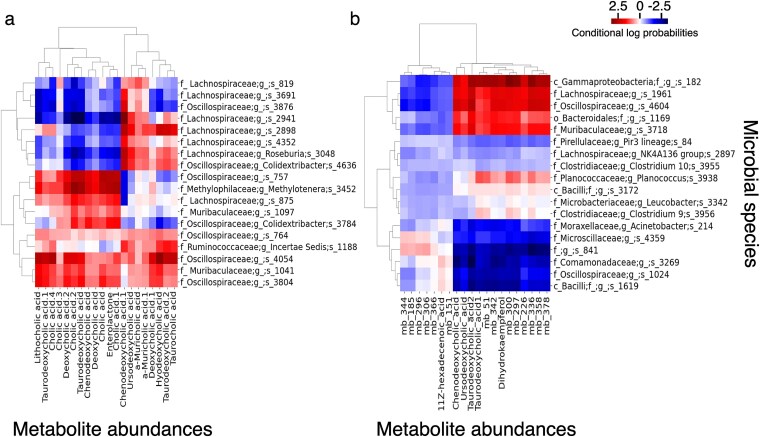
Heat map of the estimated conditional log probabilities of VBayesMM for the selected microbial species and metabolite abundances in dataset A. Individual metabolites and microbiomes were hierarchically clustered (Ward’s method) using Euclidean distance. (a) IHH cases group. (b) controls group. Note: f denotes family; g denotes genus; s denotes species; mb denotes metabolite.

To evaluate the probabilistic relationships between keystone microbial species and metabolite abundances in IHH case and control groups within dataset A, we initially identified the top 50 microbial species using $\boldsymbol{\tilde \gamma }$ value thresholds of over 0.75 for IHH cases and over 0.8 for control groups. We then calculated conditional log-probabilities between these selected microbial species and all metabolite abundances. To create a more interpretable visualization that highlights the probabilistic relationships we ranked these conditional probabilities by their absolute values. The subset of microbial species and metabolites displayed in Figs 5a and 5b shows those with the top conditional log probability values. For hierarchical clustering, we treated each row (or column) of this conditional log-probability matrix as a feature vector and computed Euclidean distances between these vectors. These distance measures were then used to perform hierarchical clustering with Ward’s linkage method on both microbial species (rows) and metabolites (columns), enabling us to identify clusters of species and metabolites with similar co-occurrence patterns. Supplementary Figure S3 presents comprehensive heatmaps of microbial species and metabolites, with hierarchical clustering initially performed on the IHH cases (Supplementary Figure S3a) and subsequently applied to the control group (Supplementary Figure S3b). This figure visualizes a global view of interaction changes between IHH cases and control groups. Additionally, Supplementary Figure S4 presents the co-occurrence probability estimates generated using MMvec for IHH cases and control groups.

Figure 5 illustrates the strength of probabilistic associations between various microbial species and specific metabolites. For example, Fig. 5a highlights that microbial families such as *Oscillospiraceae*, *Muribaculaceae*, and *Methylophilaceae* are prevalent in environments with high concentrations of cholic acid, indicating robust co-occurrence probabilities under IHH exposure. Similarly, a prominent presence of *Lachnospiraceae* in environments rich in chenodeoxycholic acid correlates with their high co-occurrence probabilities. These findings are consistent with previous research, and also expand our understanding of microbial interactions with metabolites in OSA scenarios [50, 74–76].

In particular, interaction between the *Lachnospiraceae* family and chenodeoxycholic acid appears to be a key factor in the metabolic implications in OSA. The *Lachnospiraceae*, known for their role in converting primary to secondary bile acids, may experience functional changes due to OSA-related disruptions in gut barrier function. Such disruptions could contribute to systemic inflammation and affect the microbial balance in the gut, potentially impacting bile acid metabolism [74, 75]. Chenodeoxycholic acid, a primary bile acid, plays roles not only in bile production but also as a signaling molecule [75]. It is crucial in pathways linked to inflammation and oxygen sensing, which are highly relevant to the pathophysiology of OSA. The fluctuating oxygen levels characteristic of OSA may trigger metabolic disturbances with chenodeoxycholic acid modulating the body’s response to intermittent hypoxia. These interactions underscore the importance of understanding biochemical dynamics for better management of OSA and its metabolic implications.

Further analysis using the VBayesMM approach on dataset B (Supplementary Figure S8) focused on selected probabilistic relationships between microbes and metabolites in an HFD study. This analysis revealed an association between the *Lachnospiraceae* family and hyodeoxycholic acid (HDCA) [77, 78]. As was noted earlier, the *Lachnospiraceae* family plays an important part in bile acid metabolism, by converting primary bile acids into secondary forms. HDCA is one such secondary bile acid and is synthesized from chenodeoxycholic acid via bacterial enzymatic activity within the gut. Under an HFD, the abundance of the *Lachnospiraceae* family increases – a shift that closely correlates with variations in HDCA levels [78]. This relationship emphasizes the transformative role of *Lachnospiraceae* in modulating bile acid profiles, which is pivotal to understanding dietary impacts on metabolic health. These findings offer insights into how dietary fats influence microbial dynamics and their subsequent effects on host metabolism.

### Ablation study

To evaluate the contribution of the spike-and-slab strategy and its hyperparameters we conducted the following ablation studies using dataset A.

#### Impact of hyperparameters on the predictive performance

**Figure 6:**
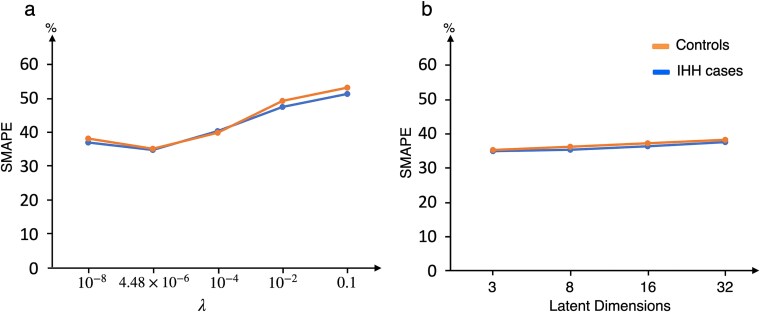
The SMAPE values for VBayesMM vary depending on the hyperparameter settings in dataset A. (a) the value of the hyperparameter $\lambda $. (b) the number of latent dimensions.

We evaluated VBayesMM’s predictive performance under different values of the hyperparameter $\lambda $ while fixing the number of latent dimensions at 3. In Dataset A, which includes 4702 taxonomic units, 92 IHH-case samples, and 90 control samples, we determined that the optimal value of $\lambda $ (denoted by $\lambda _{opt}$) was $\simeq $$4.48 \times 10^{-6}$. We compared several $\lambda $ values, including $10^{-8}, 10^{-4}, 10^{-2}$, and 0.1 [37]. As shown in Fig. 6a, when $\lambda = 10^{-8}$, the SMAPE was 36.88% (IHH cases) and 38.02% (controls), suggesting the model may be over-sparsified and is omitting useful microbial species. As $\lambda $ rose toward $\lambda _{opt} = 4.48 \times 10^{-6}$, the SMAPE reached its lowest point [34.74% (IHH cases) and 35.05% (controls)], indicating an optimal balance between dismissing irrelevant variables and retaining crucial predictors. Beyond this optimal value, SMAPE increased steadily, reaching 51.16% (IHH cases) and 53.01% (controls) at $\lambda =0.1$, suggesting that an overly large probability of feature inclusion leads to overfitting and reduced predictive accuracy. Thus, the $\lambda _{opt}$ consistently yielded the most favorable trade-off between sparsity and accuracy.

We also assessed how different values of the latent dimensions influenced the predictive performance of VBayesMM when $\lambda _{opt}$ was applied. Specifically, we tested 3, 8, 16, and 32 latent dimensions. As shown in Fig. 6b, the SMAPE values remained nearly unchanged across these settings, varying only slightly between about 35% and 38%. This suggests that increasing the number of latent dimensions beyond 3 does not meaningfully improve predictive accuracy for VBayesMM. Therefore, selecting fewer latent dimensions may be preferable, as it simplifies the model while maintaining comparable accuracy.

#### Impact of spike-and-slab strategy on the predictive performance

**Figure 7:**
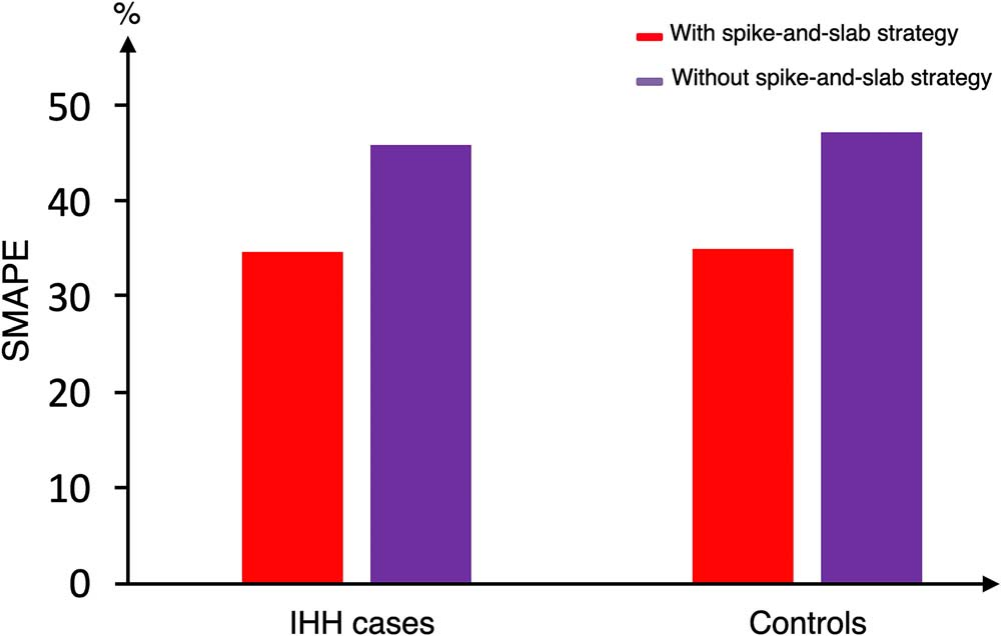
The SMAPE values are computed for the two approaches of VBayesMM in IHH case and control groups of dataset A.

We evaluated VBayesMM’s predictive performance with and without the spike-and-slab strategy, using default hyperparameter settings (Fig. 7). When the spike-and-slab approach was used, VBayesMM produced lower SMAPE values compared with the model without this prior. Specifically, the SMAPE values for the IHH cases decreased from 45.96% to 34.73%, and similarly decreased from 47.32% to 35.06% for the control group. To examine whether all microbial species contribute equally to model performance, we applied VBayesMM without the spike-and-slab approach but restricted the input to only the top 50, 100, and 200 species previously identified using the spike-and-slab approach ($\boldsymbol{\tilde \gamma }> 0.76, 0.65$, and 0.62, respectively). As illustrated in Supplementary Figure S9, the VBayesMM model based on just the top 50 species, achieved SMAPE values of 35.69% for IHH cases. The SMAPE increased slightly to 38.87% with 100 species, and 40.01% with 200 species. Similarly, the control group exhibited a similar pattern, with SMAPE values of 36.11% (top 50 species), 39.56% (top 100 species), and 40.87% (top 200 species). The observed increase in error rates when more species were retained (from 50 to 100 and 200) indicates that feature selection helps to address the curse of dimensionality. These results demonstrate that the spike-and-slab approach allows filtering out of uninformative features, leading to better predictive performance.

## Discussion

Accurate identification of core taxonomic units within the microbiome is essential for enhancing our understanding of how it relates to other omics data, most importantly from the perspective of microbe–metabolite interactions. The approach proposed in this paper aims to improve the quality of such inferences and open up new research opportunities in exploring the role microbiome plays across diverse fields such as human disease, precision medicine, and environmental studies.

The VBayesMM method represents a significant advancement in microbiome multiomics analysis, addressing the challenges posed by high dimensionality of integrated datasets, which often led to scalability issues, reduced performance, and interpretation challenges when analyzed with previous methods. VBayesMM employs a spike-and-slab prior regularization on the weight matrix and bias vector of the encoder neural network, allowing for the probabilistic identification of a limited number of taxonomic units statistically found to improve model performance. At the same time, variational inference strategy is used to address the computational bottlenecks associated with traditional Bayesian sampling algorithms to ensure both timely execution and improved predictive accuracy. The scalability of VBayesMM is demonstrated by successfully applying it to the extensive microbiome datasets derived from shotgun metagenomic sequencing [51, 52].

Recent studies support the use of neural networks for classifying microbial features and predicting host phenotypes solely from shotgun metagenomics datasets [79, 80]. VBayesMM facilitates the discovery of probabilistic relationships between metabolites and the microbiome’s community structure and function, offering valuable insights for understanding their impact on human health and disease. Additionally, VBayesMM incorporates a Bayesian neural network that quantifies uncertainties, enabling detailed probabilistic interpretations of predicted outcomes. This capability allows for the estimation of variational posterior probabilities across microbes and metabolites. The interpretability and scalability of VBayesMM facilitate its application across diverse datasets, making it a valuable tool for integrating various microbiome datasets and supporting multiomics analysis in complex biological systems.

In addition to the 16S rRNA and shotgun metagenomic sequencing and metabolome datasets currently used, microbial community metabolome (”meta-metabolomics”) datasets may provide further insights into the chemical composition and functionality of microbial ecosystems [81, 82]. Access to these expanded data types also enables a more systematic exploration of potential therapeutics and precision dietary interventions [83]. However, microbiome-derived metabolite datasets often have very high complexity and heterogeneity. When a substantial proportion of microbial gene families is uncharacterized, it becomes challenging to establish definitive connections between specific compounds and their underlying biochemical pathways. Additionally, uncertainty remains about whether a particular metabolite originates from the host, the microbiome, or external sources (e.g. the environment). Future research will integrate meta-metabolomics datasets to further assess VBayesMM’s predictive performance while also guiding the development of Bayesian methods capable of handling large-scale, noisy, and uncertain data. This direction aims to refine our knowledge of gene function characterization and enhance understanding of host–microbiome interactions.

While we proposed several solutions to address critical challenges in microbiome multiomics analysis, VBayesMM is not without limitations. The model is primarily designed to optimize the contributions of microbiome data, which is optimal for its primary analytical objectives. However, its stability and performance can be compromised when the number of metabolite abundances significantly exceeds the number of microbial species, such as in the dataset from the HFD study (dataset B). An additional limitation of VBayesMM is its sensitivity to sample heterogeneity across different metabolic subgroups. To understand how this situation can affect the stability and accuracy of the VBayesMM we have performed an additional, more challenging evaluation where metabolome abundances were clustered (using Ward’s linkage with a distance threshold of 268.58) into five distinct groups for cross-validation of dataset A. These results are included in Supplementary Table S3. We observed decreased predictive performance in mean SMAPE values for both IHH cases (42.41% $\pm $ 2.63%) and controls (43.77% $\pm $ 2.81%). These results indicate that the prediction accuracy of VBayesMM varies with different data structures. We plan to investigate strategies for improving the robustness of VBayesMM in future work. VBayesMM also treats taxonomic units as independent entities. This assumption stems from current taxonomically independent clustering methodologies in targeted gene sequencing, which rely on arbitrary sequence similarity thresholds (e.g. 97% threshold for 16S rRNA) to group sequencing reads into OTUs without considering taxonomic relationships [20, 84, 85]. Future work should address both the dimensionality challenges of mass spectrometry metabolomics datasets and incorporate phylogenetic tree structured microbiome data while maintaining computational feasibility.

While VBayesMM provides probabilistic interpretation and visualization for a subset of microbial species and metabolite profiles, holistic biological interpretation remains challenging. Many detected features may remain unannotated due to lack of matching reference metabolites in existing databases. Integration of more comprehensive omics analyses has become increasingly relevant, such as those combining metatranscriptomics and metabolomics or metaproteomics and metabolomics [86–88]. When integrating multiple omics data types, the dimensionality and computational requirements increase considerably. This complexity and the need for probabilistic approaches present significant challenges for standard personal computers. While currently we combine the variational inference and parallel computational techniques to overcome large-scale problems of the Bayesian approach, VBayesMM still requires more runtime than non-Bayesian approaches such as MiMeNet, particularly for the shotgun metagenomic sequencing studies (datasets C and D). To address these limitations, future enhancements of VBayesMM should focus on reducing computational burden and improving data integration across various omics technologies, with the goal of more effectively pinpointing the relationships between microbes and potential dietary or therapeutic agents. By improving scalability and interoperability, VBayesMM can be made more broadly applicable across diverse biological datasets and contribute to a deeper understanding of microbiome-related health dynamics.

**Key Points**

- Human microbiome studies continually reveal novel associations between microbiome compositions and disease states that allow potential applications in the development of human microbiome-related diagnostics and therapeutic strategies.
- We propose a novel method to support this type of analysis (VBayesMM), which combines deep learning with Bayesian inference to discover microbe–metabolite connections. Notably, it allows quantification of their predictive uncertainty, which is not possible with pure deep learning methods.
- Our results demonstrate that VBayesMM substantially outperforms existing methods and delivers improved run-time while enhancing the biological interpretability. The method has broad transferrable utility for possible applications in numerous other settings beyond microbiome analysis.
- Our framework facilitates the analysis of complex biomedical multiomics data and can be valuable for improving our understanding of human diseases and discovery of better treatments.

## Supplementary Material

2025_06_09_BIB_journal_VBayesMM_Supplementary_bbaf300

## Data Availability

The software VBayesMM is available at https://tungtokyo1108.github.io/VBayesMM/.
