# Supplementary material for: VBayesMM: variational Bayesian neural network to prioritize important relationships of high-dimensional microbiome multiomics data
Source: Brief Bioinform. 2025 Jul 4;26(4):bbaf300. doi: 10.1093/bib/bbaf300 (PMC12231592; doi:10.1093/bib/bbaf300)
Supplement: 2025_06_09_BIB_journal_VBayesMM_Supplementary_bbaf300 [file 2025_06_09_bib_journal_vbayesmm_supplementary_bbaf300.pdf]

Supplementary Information

VBayesMM: Variational Bayesian neural network to prioritize important relationships of high-dimensional microbiome multiomics data

Tung Dang [1], Artem Lysenko [1,2,*], Keith A. Boroevich [2] and Tatsuhiko Tsunoda [1,2,3,*]
[1] Laboratory for Medical Science Mathematics, Department of Biological Sciences, School of Science, The University of Tokyo, Tokyo, Japan
[2] Laboratory for Medical Science Mathematics, RIKEN Center for Integrative Medical Sciences, Yokohama, Japan
[3] Laboratory for Medical Science Mathematics, Department of Computational Biology and Medical Sciences, Graduate School of Frontier Sciences, The University of Tokyo, Tokyo, Japan
[*] Corresponding author(s):tsunoda@bs.s.u-tokyo.ac.jp; alysenko@g.ecc.u-tokyo.ac.jp

1 Supplementary Figures

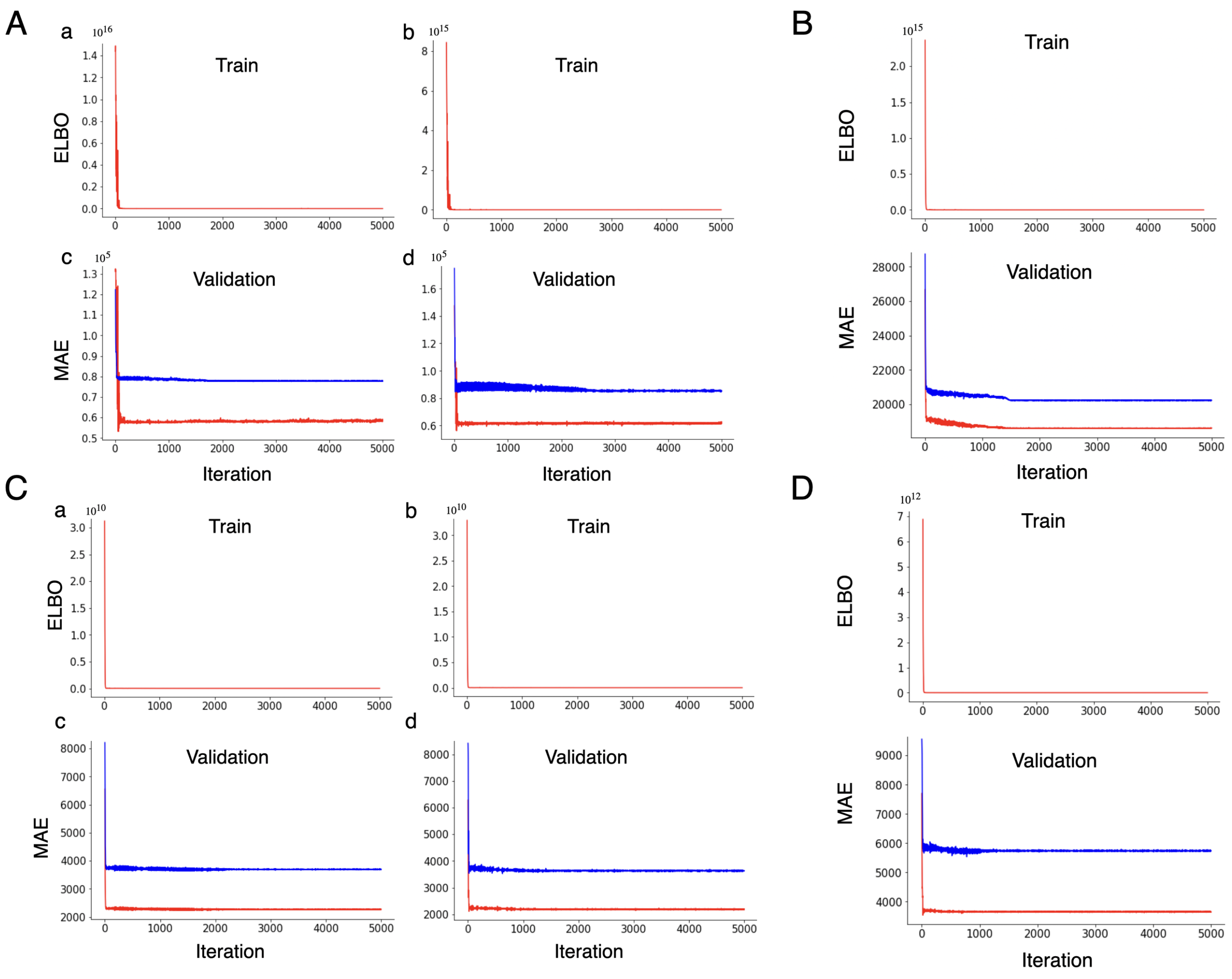

Supplementary Figure S1: The Evidence Lower Bound (ELBO) and Mean Absolute Error (MAE) values for the VBayesMM and mmvec approaches, represented in red and blue respectively. The ELBO is computed using 80% of the total samples for training, while the MAE is computed using 20% of the samples for testing. Panel A (Dataset A): Displays ELBO values for the intermittent hypoxia and hypercapnia (IHH) case and air control in parts (a) and (b), and MAE values in parts (c) and (d); Panel B (Dataset B): Shows the ELBO value in part (a) and the MAE value in part (b); Panel C (Dataset C): Features ELBO values for the gastric cancer (GC) case and healthy control in parts (a) and (b), with corresponding MAE values in parts (c) and (d); Panel D (Dataset D): Contains the ELBO value in part (a) and the MAE value in part (b).

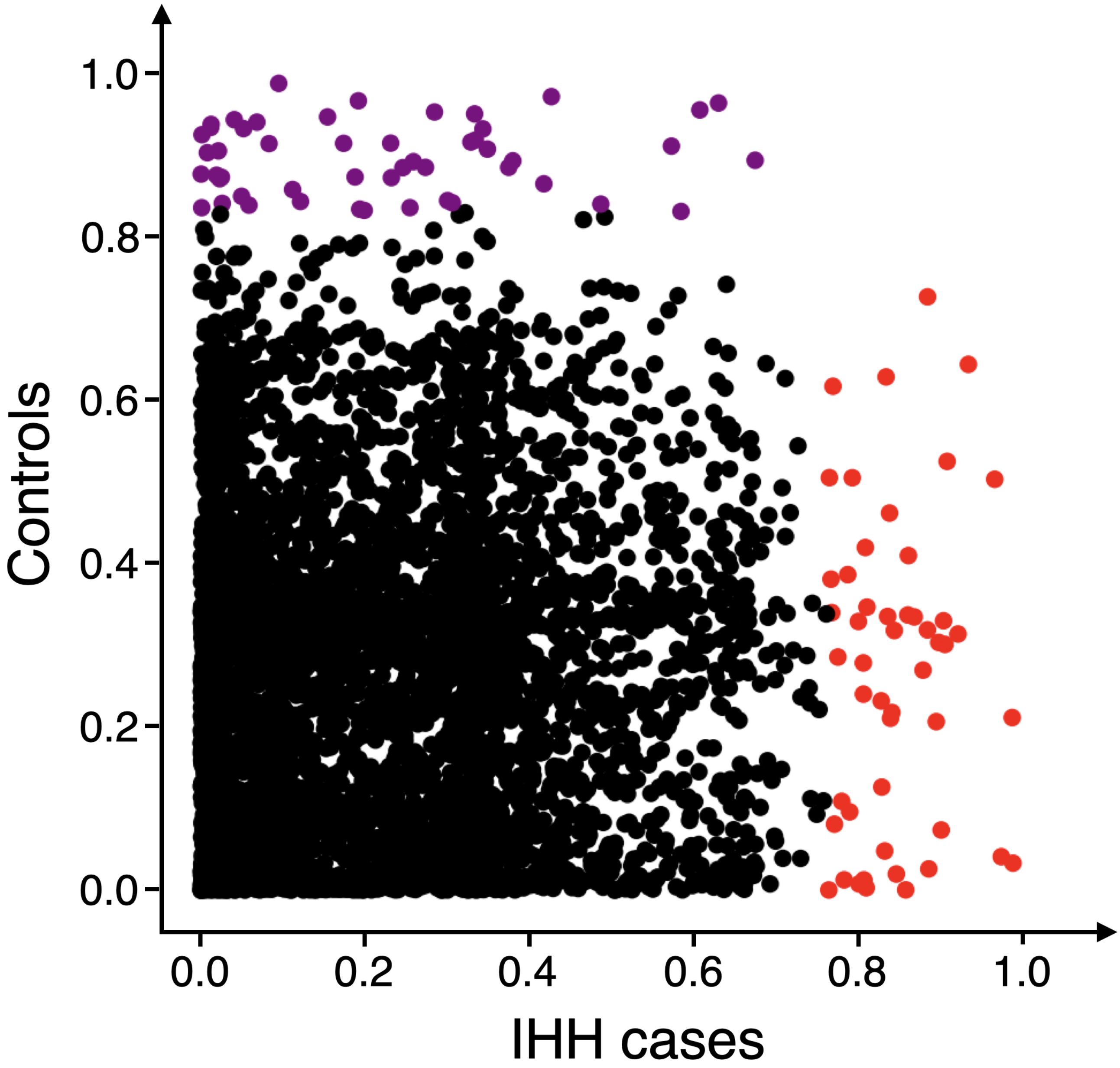

Supplementary Figure S2: Scatter plot of the average of $\tilde{\gamma} = \frac{\sum_{l=1}^{L} \gamma_{il}}{L}$ in intermittent hypoxia and hypercapnia (IHH) cases and controls group of dataset A. The top 50 microbial species identified by VBayesMM and associated with IHH cases appear in red, while the top 50 species associated with the control group appear in purple.

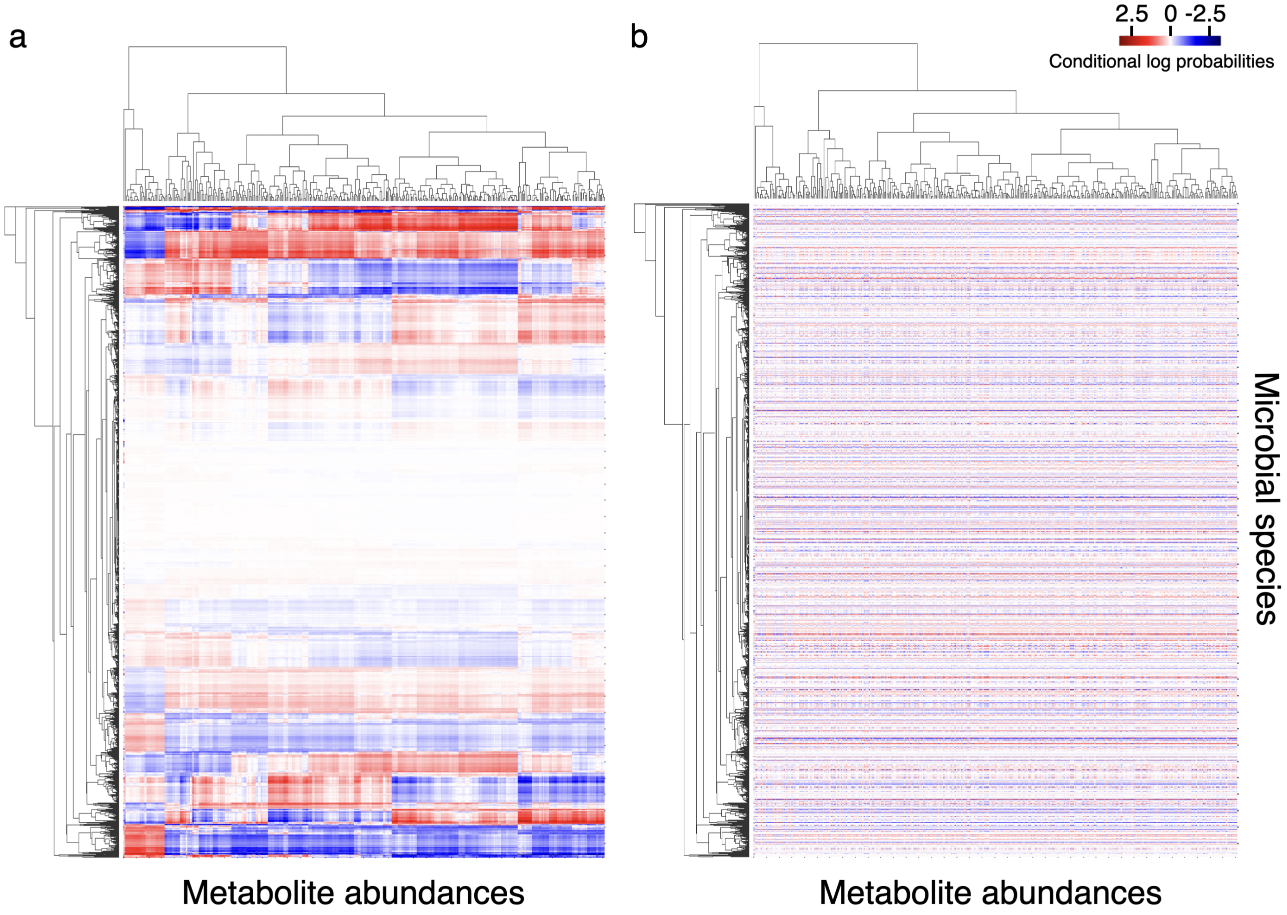

Supplementary Figure S3: Heatmap of the estimated conditional log probabilities of VBayesMM for all microbial species and metabolite abundances in dataset A. Individual metabolites and microbiomes were hierarchically clustered (Ward's method) using Euclidean distance. a. intermittent hypoxia and hypercapnia (IHH) cases group; b. controls group with the hierarchical clustering of IHH cases.

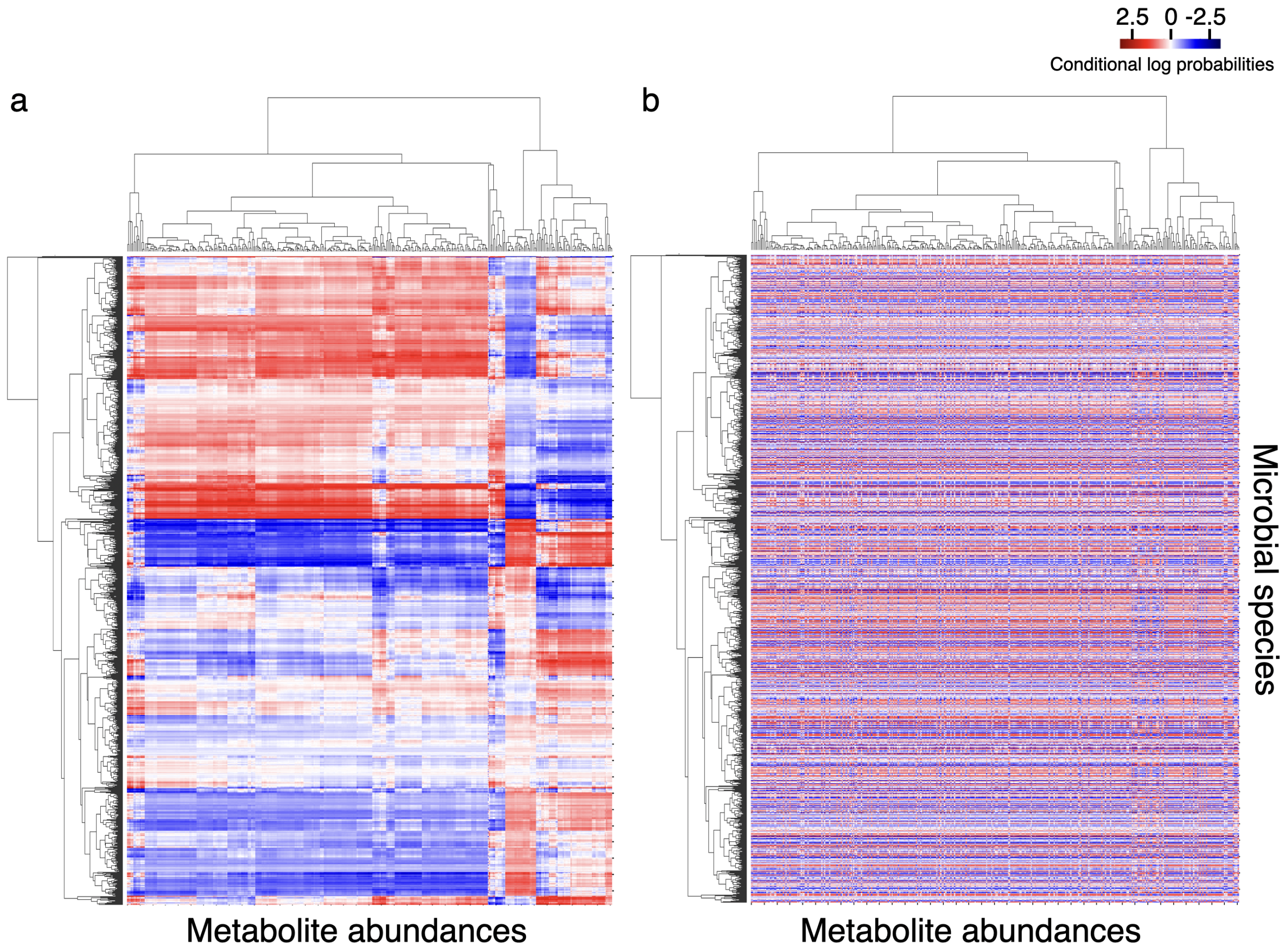

Supplementary Figure S4: Heatmap of the estimated conditional log probabilities of MMvec for all microbial species and metabolite abundances in dataset A. Individual metabolites and microbiomes were hierarchically clustered (Ward's method) using Euclidean distance. a. intermittent hypoxia and hypercapnia (IHH) cases group; b. controls group with the hierarchical clustering of IHH cases.

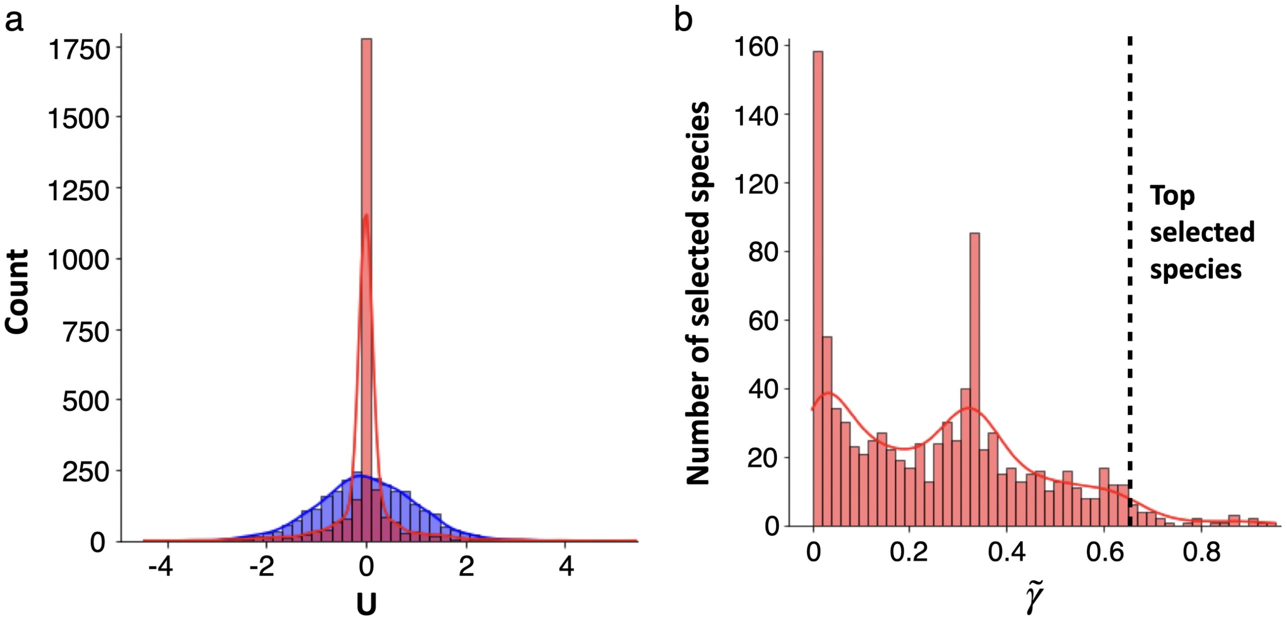

Supplementary Figure S5: Histogram of the posterior probability distribution **U** and the average of $\tilde{\gamma} = \frac{\sum_{l=1}^{L} \gamma_{il}}{L}$ in dataset B. The VBayesMM and mmvec approaches are represented in red and blue respectively. The dashed lines are bound to select microbiome species.

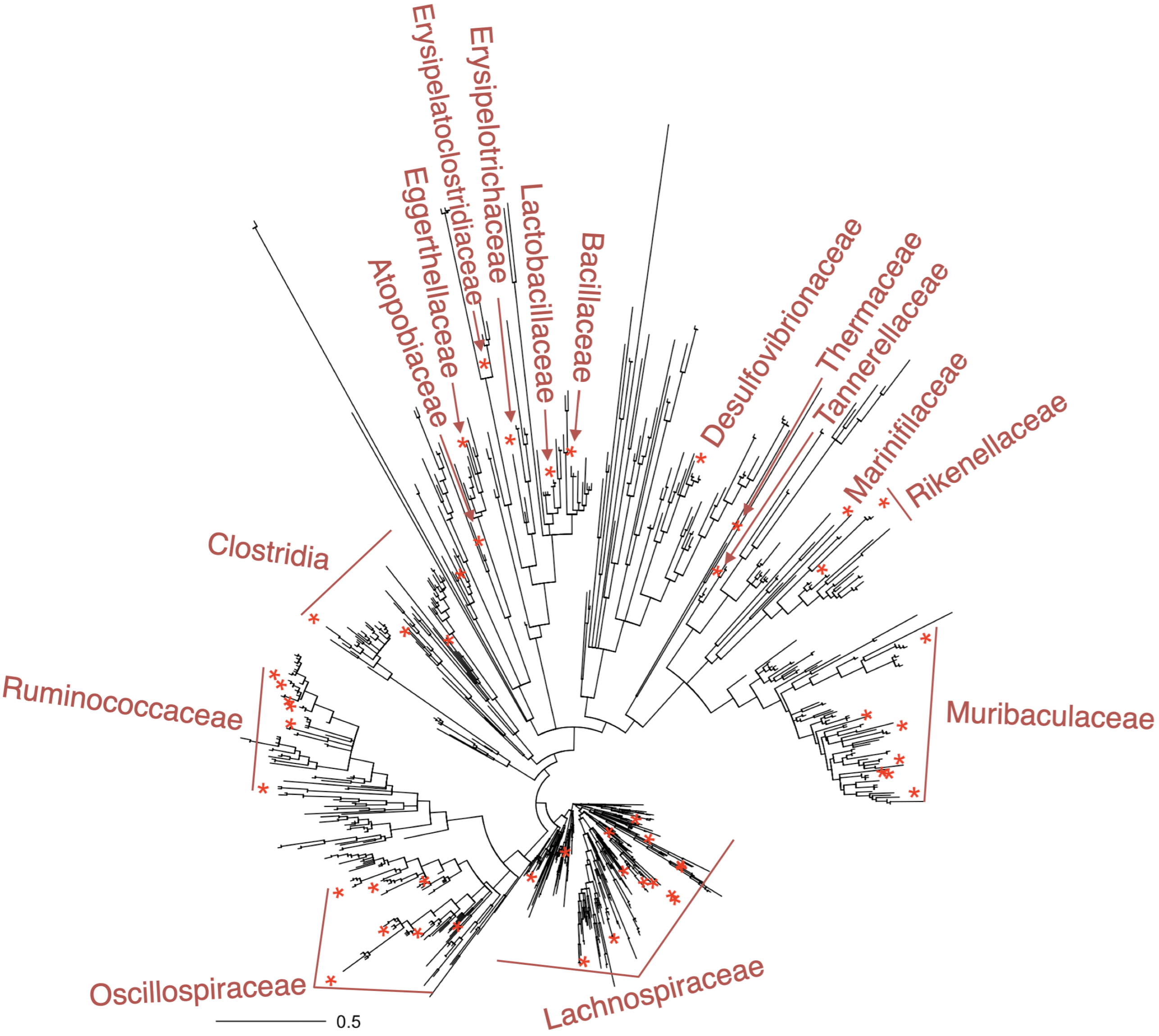

Supplementary Figure S6: Microbial species selected using the VBayesMM approach and mapped on the phylogenetic tree based on 16S rRNA gene sequences for dataset B.

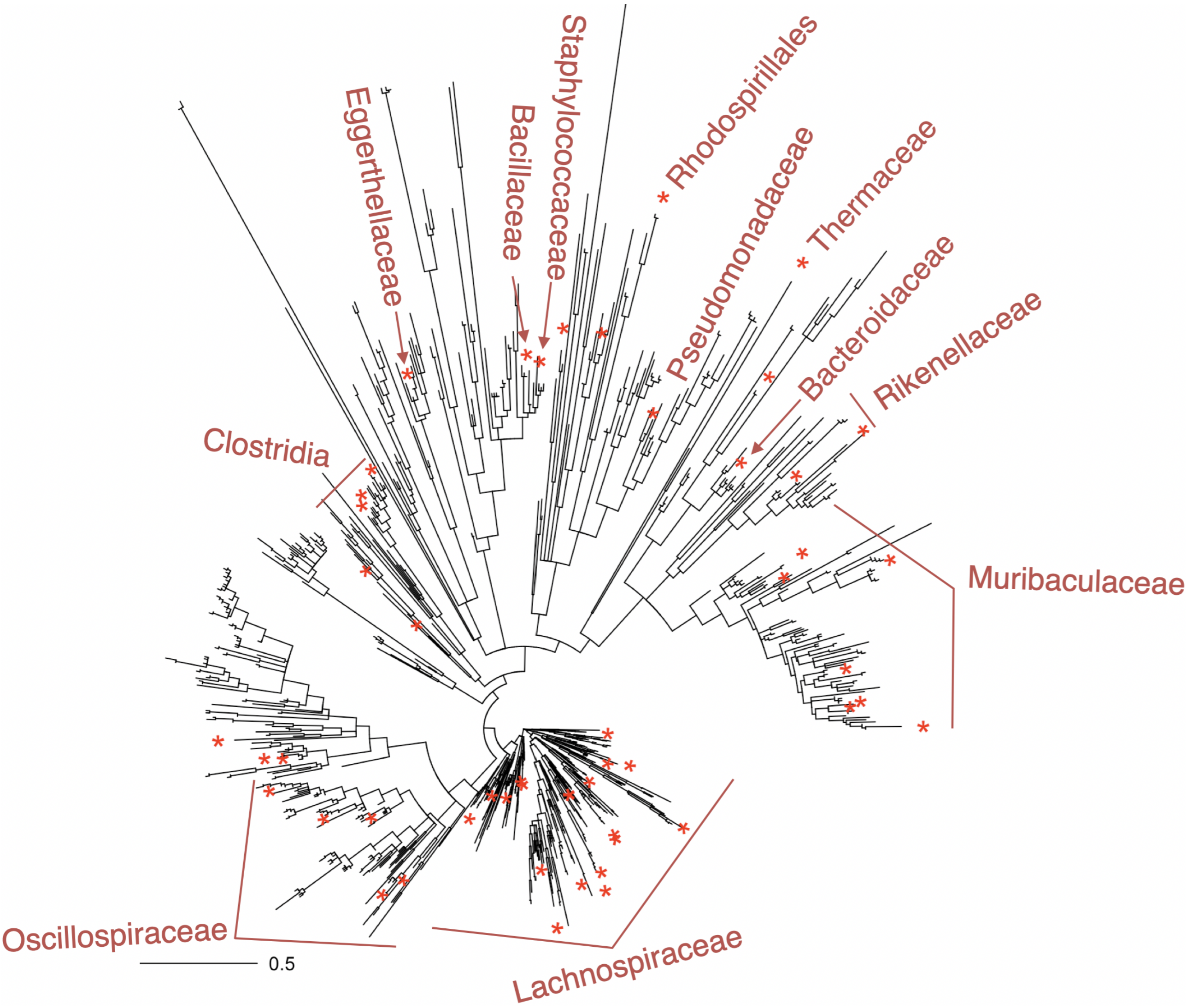

Supplementary Figure S7: Microbial species selected using the MMvec approach and mapped on the phylogenetic tree based on 16S rRNA gene sequences for dataset B.

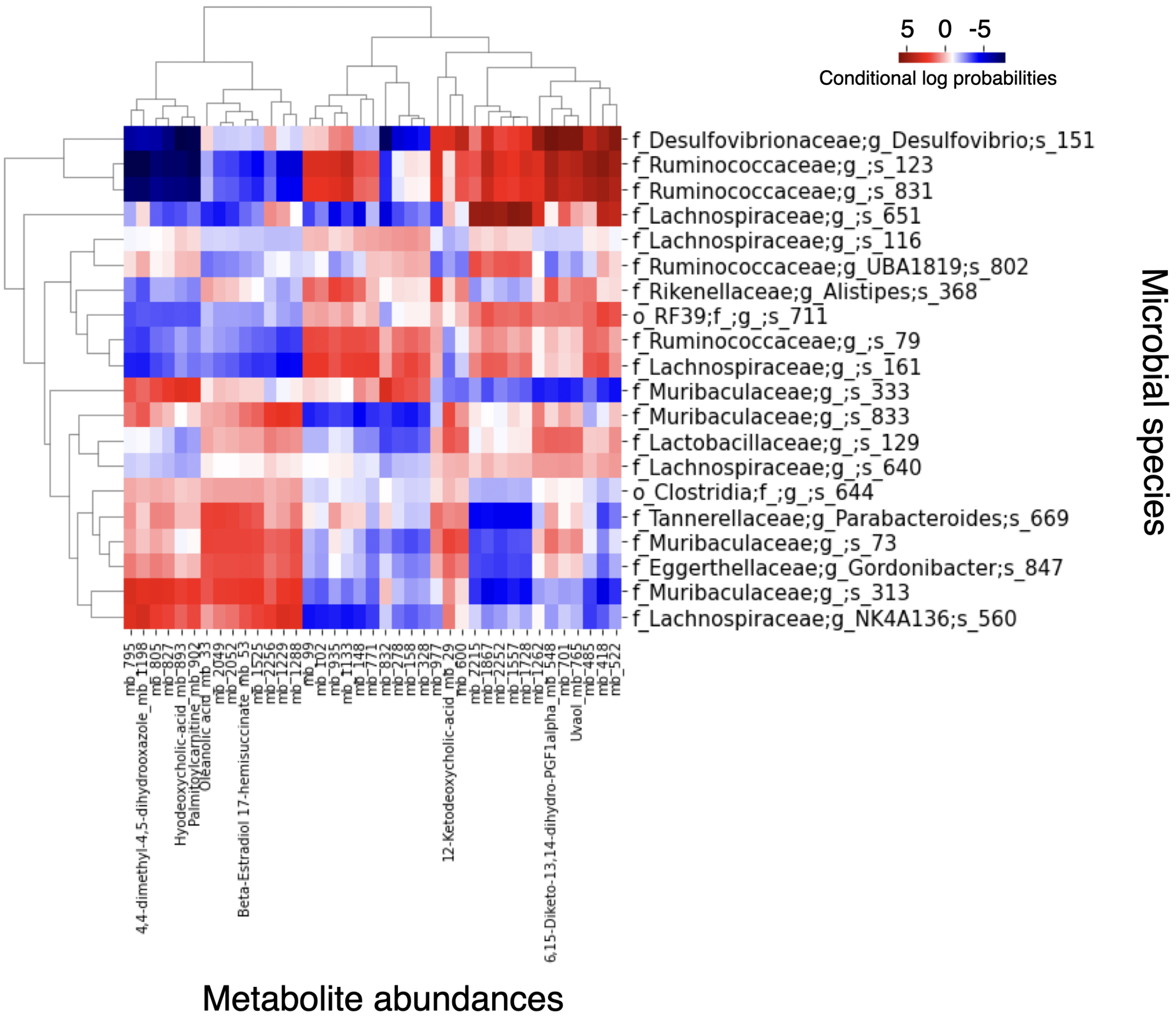

Supplementary Figure S8: Heat map of the estimated conditional log probabilities of VBayesMM for the selected microbial species and metabolite abundances in dataset B. Individual metabolites and microbiomes were hierarchically clustered (Ward's method) using Euclidean distance. Note: f denotes family; g denotes genus; s denotes species; mb denotes metabolite.

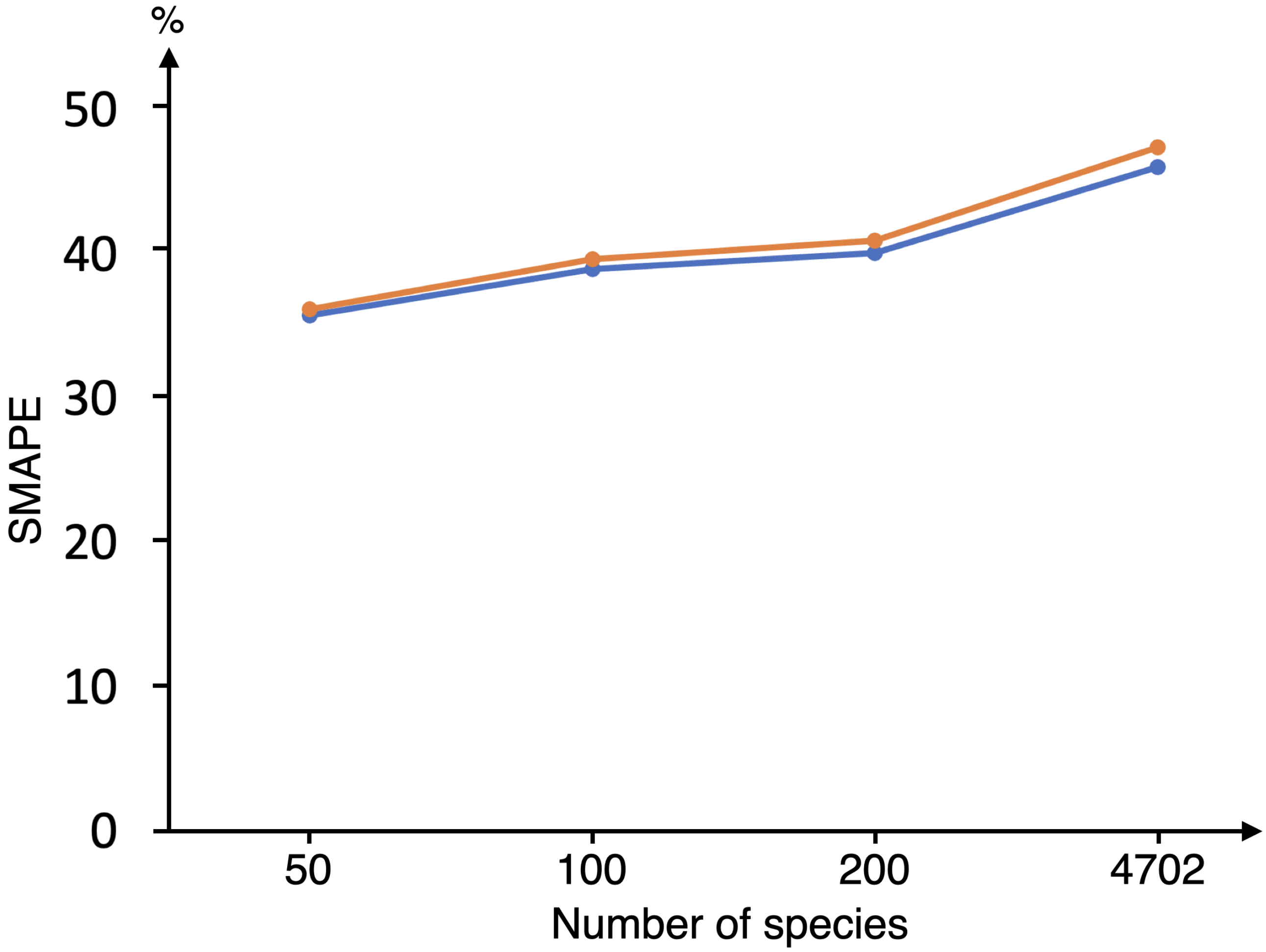

Supplementary Figure S9: The Symmetric Mean Absolute Percentage Error (SMAPE) values for VBayesMM without the spike-and-slab strategy in Dataset A. The model was evaluated using inputs consisting of the top 50, 100, and 200 species previously identified with the spike-and-slab approach, as well as all species. The intermittent hypoxia and hypercapnia (IHH) case and control groups are represented in blue and orange, respectively.

2 Supplementary Tables

Supplementary Table S1: The SMAPE values of the four approaches on the real data sets.

Dataset	Case–control	VBayesMM	MiMeNet	MMvec	sPLS
A	IHH case	34.73 %	38.82 %	47.59 %	67.52 %
	Control	35.06 %	40.11 %	48.17 %	69.37 %
B	HFD case	55.07 %	56.88 %	60.77 %	78.32 %
C	GC case	44.42 %	52.03 %	71.58 %	88.75 %
	Control	46.31 %	53.81 %	72.79 %	90.03 %
D	CRC case	48.64 %	60.06 %	76.45 %	93.12 %

Note: SMAPE: symmetric mean absolute percentage error. IHH: intermittent hypoxia and hypercapnia. HFD: high-fat die. GC: gastric cancer. CRC: colorectal cancer. All algorithms were run on a personal computer (Intel® Xeon® Gold 6230 Processor 2.10 GHz × 2, 40 cores, 2 threads per core) under Ubuntu 22.04.4 LTS.

Supplementary Table S2: Running time of the four approaches on the real data sets.

Dataset	Case–control	VBayesMM	MiMeNet	MMvec	sPLS
A	IHH case	1.52 h	1.23 h	1.48 h	2.63 h
	Control	1.50 h	1.21 h	1.45 h	2.58 h
B	HFD case	8.54 h	6.92 h	8.28 h	10.16 h
C	GC case	48.66 h	39.41 h	48.25 h	51.19 h
	Control	48.82 h	39.54 h	48.43 h	51.51 h
D	CRC case	121.62 h	98.51 h	120.97 h	125.18 h

Note: The VBayesMM, MiMeNet and MMvec packages are implemented in parallel using Python. The mixOmics package is implemented in parallel using R.

Supplementary Table S3: The mean SMAPE (standard deviation) values of VBayesMM with the spike-and-slab approach by clustering metabolome abundances into five distinct groups for cross-validation of dataset A.

Dataset	Case–control	VBayesMM with spike-and-slab
A	IHH case	42.41 % (2.63)
	Control	43.77 % (2.81)

3 Supplementary Methods

3.1 Variational inference for Variational Bayesian microbiome multiomics (VBayesMM) approach

We expand specifically the Evidence Lower Bound (ELBO) equation as follows:

$$
\begin{aligned}
&\mathcal{L}\left[q\left(\Xi|\Theta\right)\right] = \mathrm{E_q}\left[\log\left(\mathrm{p}\left(\Xi,\mathbf{D}\right)\right)\right] - \mathrm{E_q}\left[\log\left(\mathrm{q}\left(\Xi|\Theta\right)\right)\right]\\
&= \mathrm{E_q}\left[\log\left(\mathrm{p}\left(\mathbf{D}\right)\right)\right] - \mathrm{KL}\left[\mathrm{q}\left(\boldsymbol{V}\right)\|\mathrm{p}\left(\boldsymbol{V}\right)\right] - \mathrm{KL}\left[\mathrm{q}\left(\boldsymbol{\gamma}\right)\|\mathrm{p}\left(\boldsymbol{\gamma}\right)\right] - \mathrm{q}\left(\boldsymbol{\gamma}=1\right)\mathrm{KL}\left[\mathcal{N}\left(\boldsymbol{\alpha}_{\mathbf{U}},\boldsymbol{\beta}^2_{\mathbf{U}}\right)\|\mathcal{N}\left(0,\boldsymbol{\beta}^2_{0\mathbf{U}}\right)\right]\\
&= \mathrm{E_q}\left[\log\left(\mathrm{p}\left(\mathbf{D}\right)\right)\right] - 0.5\sum_{\mathrm{j=1}}^{\mathrm{M}}\sum_{\mathrm{l=1}}^{\mathrm{L}}\left[\left(1+\log\left(\beta^2_{\mathrm{V_{jl}}}\right)-\alpha^2_{\mathrm{V_{jl}}}-\beta^2_{\mathrm{V_{jl}}}\right)\right] - 0.5\sum_{\mathrm{j=1}}^{\mathrm{M}}\left[\left(1+\log\left(\beta^2_{\mathrm{V_{j0}}}\right)-\alpha^2_{\mathrm{V_{j0}}}-\beta^2_{\mathrm{V_{j0}}}\right)\right]\\
&-\sum_{i=1}^{N}\sum_{l=1}^{L}\left[\left(\tfrac{1}{1+e^{-\xi_{U_{il}}}}\right)\times\left(\log\left(\tfrac{1}{1+e^{-\xi_{U_{il}}}}\right)-\log\left(\lambda_{U_{il}}\right)\right)+\left(1-\tfrac{1}{1+e^{-\xi_{U_{il}}}}\right)\times\left(\log\left(1-\tfrac{1}{1+e^{-\xi_{U_{il}}}}\right)-\log\left(1-\lambda_{U_{il}}\right)\right)\right]\\
&-\sum_{i=1}^{N}\sum_{l=1}^{L}\left[\left(\tfrac{1}{1+e^{-\xi_{U_{il}}}}\right)\times\left(\log\left(\beta^2_{0U_{il}}\right)-\log\left(\beta^2_{U_{il}}\right)+0.5\left(\alpha^2_{U_{il}}+\beta^2_{U_{il}}\right)/\beta^2_{0U_{il}}-0.5\right)\right]\\
&-\sum_{i=1}^{N}\left[\left(\tfrac{1}{1+e^{-\xi_{U_{i0}}}}\right)\times\left(\log\left(\tfrac{1}{1+e^{-\xi_{U_{i0}}}}\right)-\log\left(\lambda_{U_{i0}}\right)\right)+\left(1-\tfrac{1}{1+e^{-\xi_{U_{i0}}}}\right)\times\left(\log\left(1-\tfrac{1}{1+e^{-\xi_{U_{i0}}}}\right)-\log\left(1-\lambda_{U_{i0}}\right)\right)\right]\\
&-\sum_{i=1}^{N}\left[\left(\tfrac{1}{1+e^{-\xi_{U_{i0}}}}\right)\times\left(\log\left(\beta^2_{0U_{i0}}\right)-\log\left(\beta^2_{U_{i0}}\right)+0.5\left(\alpha^2_{U_{i0}}+\beta^2_{U_{i0}}\right)/\beta^2_{0U_{i0}}-0.5\right)\right]
\end{aligned}
\tag{1}
$$

To compute the variational expectations $E_q[.]$ in equation (1), we use the properties of exponential family distribution. If variational distributions for $q\left(\mathbf{V}|\alpha_{\mathbf{V}},\beta^2_{\mathbf{V}}\right)$ and slab component $q\left(\mathbf{U}|\alpha_{\mathbf{U}},\beta^2_{\mathbf{U}}\right)$ are Gaussian distributions, then the exponential family representations are given by [1, 2]:

$$
\begin{aligned}
q\left(\mathbf{V}|\alpha_{\mathbf{V}},\beta^2_{\mathbf{V}}\right) &= 1/(\sqrt{2}\pi)\exp\left[(\alpha_{\mathbf{V}}/\beta^2_{\mathbf{V}})\mathbf{V} - 1/(2\beta^2_{\mathbf{V}})\mathbf{V}^2 - 1/(2\beta^2_{\mathbf{V}})\alpha^2_{\mathbf{V}} - \log(\beta_{\mathbf{V}})\right]\\
q\left(\mathbf{U}|\alpha_{\mathbf{U}},\beta^2_{\mathbf{U}}\right) &= 1/(\sqrt{2}\pi)\exp\left[(\alpha_{\mathbf{U}}/\beta^2_{\mathbf{U}})\mathbf{U} - 1/(2\beta^2_{\mathbf{U}})\mathbf{U}^2 - 1/(2\beta^2_{\mathbf{U}})\alpha^2_{\mathbf{U}} - \log(\beta_{\mathbf{U}})\right]
\end{aligned}
$$

So the natural parameters and sufficient statistics of the Gaussian distributions for $\mathbf{V}$ and $\mathbf{U}$ are $\eta_{\mathbf{V}} = \left[\alpha_{\mathbf{V}}/\beta^2_{\mathbf{V}}; -1/(2\beta^2_{\mathbf{V}})\right]$, $T(\mathbf{V}) = \left[\mathbf{V};\mathbf{V}^2\right]$ and $\eta_{\mathbf{U}} = \left[\alpha_{\mathbf{U}}/\beta^2_{\mathbf{U}}; -1/(2\beta^2_{\mathbf{U}})\right]$, $T(\mathbf{U}) = \left[\mathbf{U};\mathbf{U}^2\right]$, respectively.

Similarity, if variational distribution for $q(\boldsymbol{\gamma}|\boldsymbol{\xi})$ is Bernoulli distribution, the exponential family representations are given by:

$$
q(\boldsymbol{\gamma}|\boldsymbol{\xi}) = \exp\left[\log\left(\boldsymbol{\xi}/(1-\boldsymbol{\xi})\right)\boldsymbol{\gamma} + \log\left(1-\boldsymbol{\xi}\right)\right]
$$

The natural parameters and sufficient statistics of the Bernoulli distributions for $\boldsymbol{\gamma}$ are $\eta_{\boldsymbol{\gamma}} = [\boldsymbol{\xi}/(1-\boldsymbol{\xi})]$, $T(\boldsymbol{\gamma}) = [\boldsymbol{\gamma}]$.

3.2 Reparameterization Trick

We use reparameterization trick to make the optimization of ELBO in equation (1). For the Gaussian distribution of the embedding matrix for metabolite abundances $\mathbf{V}$ that include weight matrix V_{jl} and bias vector V_{j0}, we take samples via the following differentiable bi-variate transformation [3]:

$$
\begin{aligned}
V_{jl} &= \alpha_{V_{jl}} + \beta_{V_{jl}}\epsilon_{V_{jl}}\\
V_{j0} &= \alpha_{V_{j0}} + \beta_{V_{j0}}\epsilon_{V_{j0}}
\end{aligned}
$$

where $\epsilon_{V_{jl}} \sim \mathcal{N}(0,1)$ and $\epsilon_{V_{j0}} \sim \mathcal{N}(0,1)$. Then, we calculate and update their mean and standard deviation via the optimization of ELBO that can utilize the stochastic gradient approach as follows:

$$
\begin{aligned}
\nabla_{\alpha_{V_{jl}},\beta_{V_{jl}}}\mathcal{L}\left[\alpha_{V_{jl}},\beta_{V_{jl}}\right] &= \sum_{j=1}^{M}\sum_{l=1}^{L}\left(\nabla_{\alpha_{V_{jl}},\beta_{V_{jl}}}\mathrm{E_q}\left[\log\left(\mathrm{p}\left(\mathbf{D}\right)\right)\right] - \nabla_{\alpha_{\mathrm{V_{jl}}},\beta_{\mathrm{V_{jl}}}}\mathrm{KL}\left[\mathcal{N}\left(\alpha_{\mathrm{V_{jl}}},\beta^2_{\mathrm{V_{jl}}}\right)\|\mathcal{N}\left(0,\beta^2_{0\mathrm{V_{jl}}}\right)\right]\right)\\
\nabla_{\alpha_{V_{j0}},\beta_{V_{j0}}}\mathcal{L}\left[\alpha_{V_{j0}},\beta_{V_{j0}}\right] &= \sum_{j=1}^{M}\left(\nabla_{\alpha_{V_{j0}},\beta_{V_{j0}}}\mathrm{E_q}\left[\log\left(\mathrm{p}\left(\mathbf{D}\right)\right)\right] - \nabla_{\alpha_{\mathrm{V_{j0}}},\beta_{\mathrm{V_{j0}}}}\mathrm{KL}\left[\mathcal{N}\left(\alpha_{\mathrm{V_{j0}}},\beta^2_{\mathrm{V_{j0}}}\right)\|\mathcal{N}\left(0,\beta^2_{0\mathrm{V_{j0}}}\right)\right]\right)
\end{aligned}
$$

To reparameterize the discrete variable γ, we utilized the Gumbel-softmax approximation [4] to approximate Bernoulli distribution, we take samples of spike-and-slab distribution via the following transformation:

$$
\begin{aligned}
U_{il} &= \left(1+\exp\left(-\zeta_{\mathrm{U_{il}}}/\iota\right)\right)^{-1}\left(\alpha_{U_{il}} + \beta_{U_{il}}\epsilon_{U_{il}}\right)\\
\zeta_{U_{il}} &= \log\left(\xi_{\mathrm{il}}/\left(1-\xi_{\mathrm{il}}\right)\right) + \log\left(\kappa_{\mathrm{il}}/\left(1-\kappa_{\mathrm{il}}\right)\right)\\
U_{i0} &= \left(1+\exp\left(-\zeta_{\mathrm{U_{i0}}}/\iota\right)\right)^{-1}\left(\alpha_{U_{i0}} + \beta_{U_{i0}}\epsilon_{U_{i0}}\right)\\
\zeta_{U_{i0}} &= \log\left(\xi_{\mathrm{i0}}/\left(1-\xi_{\mathrm{i0}}\right)\right) + \log\left(\kappa_{\mathrm{i0}}/\left(1-\kappa_{\mathrm{i0}}\right)\right)
\end{aligned}
$$

where $\epsilon_{U_{jl}} \sim \mathcal{N}(0,1)$, $\epsilon_{U_{j0}} \sim \mathcal{N}(0,1)$, $\kappa_{il} \sim \mathcal{N}(0,1)$, and $\kappa_{i0} \sim \mathcal{N}(0,1)$. Then, we calculate and update the variational parameters via the optimization of ELBO that can utilize the stochastic gradient approach as follows:

$$\nabla_{\alpha_{U_{jl}},\beta_{U_{jl}},\zeta_{U_{il}}}\mathcal{L}\left[\alpha_{U_{jl}},\beta_{U_{jl}},\zeta_{U_{il}}\right] = \sum_{i=1}^{N}\sum_{l=1}^{L}\left(\nabla_{\alpha_{U_{il}},\beta_{U_{il}},\zeta_{U_{il}}}\mathrm{E_q}\left[\log\left(\mathrm{p}\left(\mathbf{D}\right)\right)\right] - \nabla_{\zeta_{\mathrm{U_{il}}}}\mathrm{KL}\left[\mathrm{q}\left(\gamma\right)\|\mathrm{p}\left(\gamma\right)\right] - \nabla_{\alpha_{\mathrm{U_{jl}}},\beta_{\mathrm{U_{jl}}}}\mathrm{KL}\left[\mathcal{N}\left(\alpha_{\mathrm{U_{jl}}},\beta^2_{\mathrm{U_{jl}}}\right)\|\mathcal{N}\left(0,\beta^2_{0\mathrm{U_{jl}}}\right)\right]\right)$$

$$\nabla_{\alpha_{U_{j0}},\beta_{U_{j0}},\zeta_{U_{i0}}}\mathcal{L}\left[\alpha_{U_{j0}},\beta_{U_{j0}},\zeta_{U_{i0}}\right] = \sum_{i=1}^{N}\left(\nabla_{\alpha_{U_{i0}},\beta_{U_{i0}},\zeta_{U_{i0}}}\mathrm{E_q}\left[\log\left(\mathrm{p}\left(\mathbf{D}\right)\right)\right] - \nabla_{\zeta_{\mathrm{U_{i0}}}}\mathrm{KL}\left[\mathrm{q}\left(\gamma\right)\|\mathrm{p}\left(\gamma\right)\right] - \nabla_{\alpha_{\mathrm{U_{j0}}},\beta_{\mathrm{U_{j0}}}}\mathrm{KL}\left[\mathcal{N}\left(\alpha_{\mathrm{U_{j0}}},\beta^2_{\mathrm{U_{j0}}}\right)\|\mathcal{N}\left(0,\beta^2_{0\mathrm{U_{j0}}}\right)\right]\right)$$

References

[1] M. I. Jordan, Z. Ghahramani, T. S. Jaakkola, and L. K. Saul, "An introduction to variational methods for graphical models," *Machine learning*, vol. 37, pp. 183–233, 1999.

[2] D. M. Blei, A. Kucukelbir, and J. D. McAuliffe, "Variational inference: A review for statisticians," *Journal of the American statistical Association*, vol. 112, no. 518, pp. 859–877, 2017.

[3] D. P. Kingma, "Auto-encoding variational bayes," *arXiv preprint arXiv:1312.6114*, 2013.

[4] J. Bai, Q. Song, and G. Cheng, "Efficient variational inference for sparse deep learning with theoretical guarantee," *Advances in Neural Information Processing Systems*, vol. 33, pp. 466–476, 2020.
